# Highly Efficient Electrocatalytic N_2_ Reduction to Ammonia over Metallic 1T Phase of MoS_2_ Enabled by Active Sites Separation Mechanism

**DOI:** 10.1002/advs.202103583

**Published:** 2021-11-05

**Authors:** Ruoqi Liu, Ting Guo, Hao Fei, Zhuangzhi Wu, Dezhi Wang, Fangyang Liu

**Affiliations:** ^1^ School of Materials Science and Engineering Central South University Changsha 410083 China; ^2^ School of Metallurgy and Environment Central South University Changsha 410083 China

**Keywords:** electrocatalysis, metallic phase, molybdenum disulfide, nitrogen reduction reaction

## Abstract

The 1T phase of MoS_2_ has been widely reported to be highly active toward the hydrogen evolution reaction (HER), which is expected to restrict the competitive nitrogen reduction reaction (NRR). However, in this work, a prototype of active sites separation over 1T‐MoS_2_ is proposed by DFT calculations that the Mo‐edge and S atoms on the basal plane exhibit different catalytic NRR and HER selectivity, and a new role‐playing synergistic mechanism is also well enabled for the multistep NRR, which is further experimentally confirmed. More importantly, a self‐sacrificial strategy using g‐C_3_N_4_ as templates is proposed to synthesize 1T‐MoS_2_ with an ultrahigh 1T content (75.44%, named as CNMS, representing the composition elements of C, N, Mo, and S), which yields excellent NRR performances with an ammonia formation rate of 71.07 µg h^–1^ mg^–1^_cat._ at −0.5 V versus RHE and a Faradic efficiency of 21.01%. This work provides a promising new orientation of synchronizing the selectivity and activity for the multistep catalytic reactions.

## Graphical abstract

A prototype of active sites separation over 1T‐MoS_2_ is proposed in which the Mo‐edge and S atoms exhibit different catalytic NRR and HER selectivity. Benefiting from the active site separation, the 1T phase can accelerate both NRR and HER, but prefer the former, thus achieving the synchronization of selectivity and activity toward the NRR.

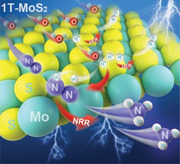

## 1 Introduction

Ammonia (NH_3_) synthesis is one of the most important processes for all life forms because it supplies valuable chemicals for the agricultural industries as well as others.^[^ ^1^, ^2^, ^3^ ^]^ To date, NH_3_ is predominantly produced by the traditional Haber‐Bosch process in which the harsh reaction conditions of high pressure and temperature are inevitably required, causing heavy energy consumption,^[^ ^2^, ^4^ ^]^ together with significant CO_2_ emission.^[^ ^5^ ^]^ Thus, an environmentally benign and sustainable alternative route for NH_3_ production is urgently desired.

The electrochemical conversion approach offers a promising cost‐efficient and environmentally friendly strategy for artificial NH_3_ synthesis at the ambient temperature and pressure, but it requires the identification of electrocatalysts with high activity for the NRR.^[^ ^6^, ^7^, ^8^, ^9^ ^]^ Among the catalysts for NRR, Mo‐based complex catalysts have emerged as promising candidates.^[^ ^10^, ^11^, ^12^, ^13^ ^]^ Besides, many efforts have been devoted to Mo‐based catalysts including single‐Mo atoms,^[^ ^14^ ^]^ molybdenum sulfides,^[^ ^15^ ^]^ molybdenum nitrides,^[^ ^16^, ^17^ ^]^ and molybdenum carbides.^[^ ^18^ ^]^ Among them, MoS_2_ plays a significant role in NRR owing to the similar constituent elements compared to natural nitrogenase.^[^ ^19^ ^]^ Sun et al. initially reported that the MoS_2_ nanosheets possessed good electrocatalytic performance toward the NRR with a NH_3_ yield of 13.09 µg h^–1^ mg^–1^_cat._ and Faradic efficiency (FE) of 1.17% under the ambient conditions.^[^ ^20^ ^]^ Then they designed defect‐rich 2H‐MoS_2_ nanoflower and obtained a boosted NH_3_ yield rate of 29.28 µg h^–1^ mg^–1^_cat._ at −0.4 V versus RHE.^[^ ^21^ ^]^ However, its catalytically inert basal planes and poor conductivity limit the large‐scale applications.^[^ ^22^ ^]^ Compared to 2H‐MoS_2_, metallic 1T‐MoS_2_ offers interesting benefits for electrocatalysis with more active sites along the basal surface as well as superior electronic conductivity.^[^ ^23^, ^24^ ^]^ Though the 1T‐MoS_2_ exhibits excellent HER performance, which serves as a competitive reaction of NRR, it is also considered as a promising NRR catalyst with amazing intrinsic catalytic activity and conductivity.^[^ ^25^, ^26^, ^27^ ^]^ Most recently, 1T MoS_2_‐based modified electrodes have been attempted as NRR electrocatalysts, such as 1T‐MoS_2_ NDs/g‐C_3_N_4_,^[^ ^28^ ^]^ 1T‐MoS_2_‐Ni,^[^ ^29^ ^]^ and SV‐1T‐MoS_2_@MoO_3_,^[^ ^30^ ^]^ all of which possess decent performances confirmed experimentally due to the existence of 1T phase. Nonetheless, the specific competition of the HER and NRR over 1T‐MoS_2_ has never been discussed, which is urgently demanded to be further investigated.

In this work, we first proposed the prototype of active sites separation over 1T‐MoS_2_ that the Mo‐edge and S atoms on the basal plane are selective to NRR and HER, respectively, which was further confirmed experimentally and theoretically. Moreover, the contents of 1T phase in MoS_2_ were regulated using g‐C_3_N_4_ nanosheets as self‐sacrificial templates, and their evolution relation with the corresponding NRR performances has been clarified.

## 2 Results and Discussion

**Figure 1:**
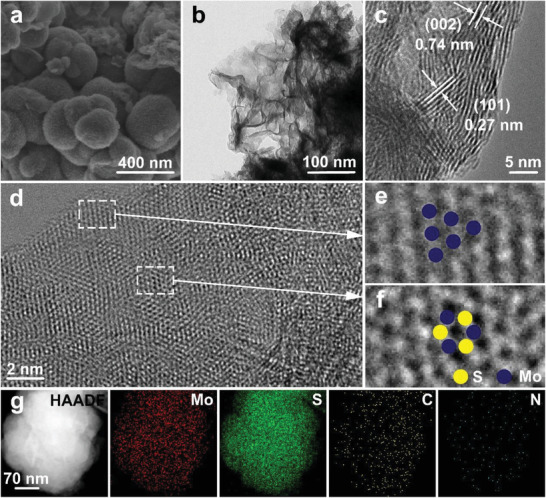
a) SEM, b) TEM, and c,d) HRTEM images of CNMS. e,f) images of the region enclosed by the white rectangle of (d). g) EDX mapping of CNMS.

The scanning electron microscopy (SEM) image and transmission electron microscopy (TEM) image (**Figure** **1**a,b) reveal well‐defined nanoflower morphology of as‐prepared CNMS. The interplanar distance is determined from the HRTEM images (Figure 1c), which is 0.74 nm, significantly larger than that of bulk 2H‐MoS_2_ (0.61 nm), implying the existence of intercalation ions or molecules.^[^ ^31^, ^32^, ^33^ ^]^ The high‐resolution TEM (HRTEM) image (Figure 1d) demonstrates the coexistence of the octahedral 1T phase and the trigonal prismatic 2H phase in CNMS, and a clear view over the detailed microstructures of 1T and 2H phases can be clearly observed from Figure 1e,f, respectively.^[^ ^34^, ^35^ ^]^ The TEM mapping images in Figure 1g affirm the uniform distribution of Mo, S, C,and N in CNMS.

**Figure 2:**
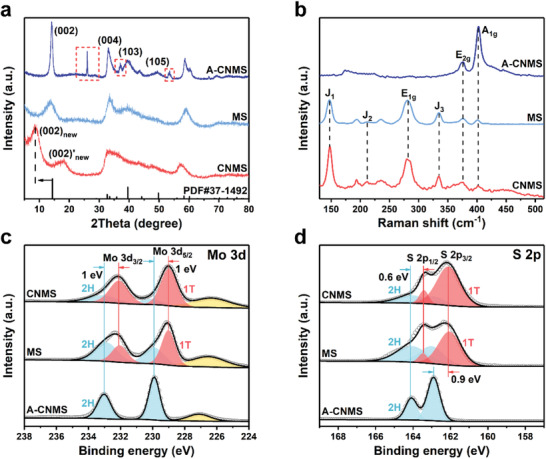
a) XRD patterns, b) Raman spectra, c) XPS spectra of Mo 3d, and d) XPS spectra of S 2p over A‐CNMS, MS, and CNMS.

**Figure** **2**a presents the XRD pattern of CNMS, in which the (002) characteristic peak separates into two diffraction peaks locating at 8.7° and 17.5°, indicating an expanded interlayer distance due to the inserted species.^[^ ^36^ ^]^ And there are no typical diffraction peaks of g‐C_3_N_4_, suggesting the complete decomposition of g‐C_3_N_4_ during the hydrothermal treatment,^[^ ^37^ ^]^ as compared in Figure S1 (Supporting Information). Besides, to verify the key role of inserted species and investigate the phase transformation, the CNMS was annealed at 800 °C for 2 h in Ar (named as A‐CNMS). As compared in Figure 2a, all the diffraction peaks can be well indexed to the standard pattern of hexagonal MoS_2_ (PDF cards: 37–1492) in A‐CNMS, which verifies that the g‐C_3_N_4_ triggers the remarkable lattice expansion. After annealing, the (002) plane of A‐CNMS moves back to 14.1°, indicating the phase transformation from metastable 1T‐MoS_2_ to thermodynamically stable 2H‐MoS_2_ with a decreased interlayer distance. Besides, several new graphite characteristic peaks appear after annealing, as illustrated in the region enclosed by the red rectangle of Figure 2a, which is due to the graphitization of inserted small carbon molecules after high‐temperature calcination.^[^ ^38^ ^]^ In addition, Fourier transform infrared spectra (FTIR) demonstrates the decomposition of the ring structure of g‐C_3_N_4_ and the formation of small carbonic molecules (Figure S2, Supporting Information), while differential scanning calorimetry‐thermogravimetric curve (DSC/TGA) further proves that carbonic molecules mentioned above are inserted into the interlayers of MoS_2_ (Figure S3, Supporting Information).

Figure 2b shows the Raman spectra, presenting three distinct peaks at 148, 212, and 335 cm^–1^ for MS and CNMS, consistent with J_1_, J_2_, and J_3_ vibrational modes of 1T‐MoS_2_.^[^ ^39^, ^40^ ^]^ The additional peak E_1g_ (282 cm^–1^) of metallic MoS_2_ can also be observed in MS and CNMS, certainly demonstrating the existence of 1T phase MoS_2_.^[^ ^19^ ^]^ In comparison, there are only two typical peaks at 376 and 402 cm^–1^ corresponding to the E_2g_ and A_1g_ vibrational modes of 2H‐MoS_2_ in A‐CNMS, demonstrating the successful conversion from 1T phase to 2H phase after calcination.^[^ ^22^ ^]^ Note that characteristic signals of 1T phase are also found over the as‐prepared MS, but are much weaker than that of CNMS. As a result, it can be concluded that the 1T phase can also be synthesized without g‐C_3_N_4_ with lower contents, which should be attributed to the insertion of other small molecules.^[^ ^30^, ^31^, ^36^ ^]^

To further verify the formation of 1T phase and determine the corresponding contents, the X‐ray photoelectron spectroscopy (XPS) spectrum of CNMS was collected, demonstrating the coexistence of Mo, S, C, and N elements (Figure S4, Supporting Information). To give a direct comparison, the Mo 3d spectra of various samples are presented in Figure 2c, and two dominant peaks of Mo 3d_3/2_ and Mo 3d_5/2_ located at 233 and 230 eV in A‐CNMS can be ascribed to the 2H phase of MoS_2_.^[^ ^15^ ^]^ As for CNMS and MS, the other two characteristic peaks at 229 and 232 eV are allocating to Mo 3d_3/2_ and Mo 3d_5/2_ of the 1T phase of MoS_2_.^[^ ^31^, ^40^, ^41^ ^]^ According to the peak area ratios of deconvoluted Mo 3d peaks, the phase contents are calculated and presented in Figure S5, Supporting Information. It can be seen that the relative content of 1T phase in CNMS reaches up to 75.44%, much larger than that of MS (40.56%), implying the vital role of the sacrificial g‐C_3_N_4_ template in the phase transformation from 2H to 1T. Besides, in Figure 2d, two peaks appearing at 163 and 164 eV are attributed to S 2p_3/2_ and S 2p_1/2_ of 2H‐MoS_2_.^[^ ^36^ ^]^ The other two peaks at binding energies around 162.1 and 163.4 eV are in agreement with the 1T‐MoS_2_, further demonstrating the formation of metallic MoS_2_.^[^ ^36^ ^]^ In addition, the element contents attained from XPS and EDX have been presented in Table S1 (Supporting Information). The results from both XPS and EDX exhibit a similar tendency: the atomic ratio of C and N is far from the theoretical value of g‐C_3_N_4_, indicating that the g‐C_3_N_4_ templates have decomposed into carbolic molecular under the hydrothermal conditions.

To clarify the operation mechanism of g‐C_3_N_4_ sacrificial templates, the effect of hydrothermal temperature was explored. In the preparation, some white flocs attached to the black MoS_2_ powders at low temperatures were observed. Therefore, the hydrothermal treatment of g‐C_3_N_4_ templates without adding molybdenum and sulfur sources was conducted at 180, 200, 220, 240, and 260 ℃ for 13 h, respectively. As depicted in Figure S6a (Supporting Information), with the increased temperatures from 180 to 220 ℃, the g‐C_3_N_4_ suspension changes to a colloidal solution, confirming that g‐C_3_N_4_ is almost completely dissolved at 220 ℃ and decomposed into small (carbonic) molecules. Ultraviolet‐visible spectra were collected to explore the constitution of the above suspension after hydrothermal reactions. As shown in Figure S6b (Supporting Information), the typical peaks of g‐C_3_N_4_ exhibit an obvious blue‐shift with the rise of hydrothermal temperature, demonstrating the molecular structure conversion of g‐C_3_N_4_.

XPS spectra of CNMS samples obtained at various hydrothermal temperatures were also collected. The Mo 3d and S 2p spectra are shown in Figure S7a,b (Supporting Information), and the phase contents were also calculated based on the former spectra and illustrated in Figure S8 (Supporting Information). With the increase of hydrothermal temperatures, the 1T phase content is gradually increased and reaches the maximum at 220 ℃ and then decreases at higher temperatures, meaning that 220 ℃ is the optimum temperature for the formation of the 1T phase. The spectra of C 1s (Figure S7c, Supporting Information) can be divided into three peaks located at 284.6 (sp^2^‐bonded carbon (C—C)), 286.3 (C—N bond), and 288.4 eV (sp^2^‐hybridized carbon (C—N═C)).^[^ ^42^, ^43^ ^]^ The N 1s spectra (Figure S7d, Supporting Information) shows typical feature peaks at 397.1, 398.3, and 399.4 eV, which can be ascribed to C—N—H, N—C_3_ and C—N═C,^[^ ^44^ ^]^ respectively. The results indicate that the g‐C_3_N_4_ is gradually dissolved with the increase of hydrothermal temperatures and almost completely dissolved at 220 ℃, which is in accordance with the digital photos (Figure S6, Supporting Information). Moreover, the XPS was also adopted to clarify the relationship between the metallic 1T phase content and the ratio of g‐C_3_N_4_/MoS_2_, and the corresponding XPS spectra and phase contents are shown in Figures S9 and S10 (Supporting Information), respectively. One can see that the content of metallic 1T phase is gradually enlarged with the increased ratio of g‐C_3_N_4_/MoS_2_ and peaks over CNMS‐5 and then drops down. Obviously, the induced formation of metallic 1T phase achieves saturation with superfluous g‐C_3_N_4_.

**Figure 3:**
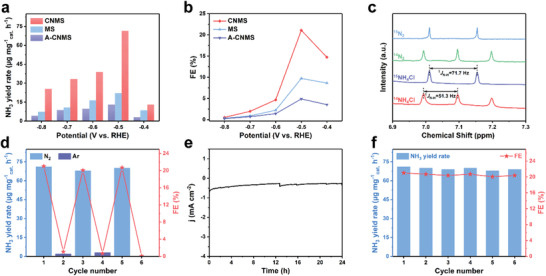
Comparison of a) NH_3_ yield rates and b) FEs for various catalysts at various potentials. c) ^1^H‐NMR (500 MHz) spectra of solution after electrocatalytic NRR using CNMS as the electrocatalyst in ^14^N_2_ or ^15^N_2_ atmosphere. d) NH_3_ yield rates and corresponding FEs of CNMS with switching 2 h cycles between N_2_‐ and Ar‐saturated electrolytes. e) Time‐dependent current density curve of CNMS at −0.5 V for 24 h. f) Stability tests of CNMS during repeated NRR at −0.5 V.

The as‐synthesized catalysts were deposited on glassy carbon electrodes and employed for the NRR test as working electrodes in a three‐compartment cell under ambient conditions, which was separated by a proton exchange membrane. The catalytic performance of as‐obtained catalysts was evaluated by measuring the generated NH_3_ via indophenol blue method, and a possible byproduct (N_2_H_4_) was also estimated by the Watt and Chrisp method. The calibration curves are given in Figures S11 and S12 (Supporting Information), respectively. **Figure** **3**a,b shows the NH_3_ yield rates and FEs of as‐obtained catalysts at various potentials. The CNMS exhibits a remarkable NH_3_ yield rate of 71.07 µg h^–1^ mg^–1^_cat._ and FE of 21.01% at −0.5 V versus RHE, much higher than that of MS (NH_3_ yield rate: 21.93 µg h^–1^ mg^–1^_cat._, FE: 9.72%) and A‐CNMS (NH_3_ yield rate: 13.08 µg h^–1^ mg^–1^_cat._, FE: 4.88%), implying that the 1T phases of MoS_2_ are main active sites for the electrocatalytic NRR. Linear sweep voltammetry (LSV) curves of CNMS in N_2_‐ and Ar‐saturated 0.1 м Na_2_SO_4_ solutions are provided in Figure S13a (Supporting Information). Note that the CNMS exhibits a higher catalytic current density under the N_2_ atmosphere, demonstrating its catalytic activity for NRR. The time‐dependent current density curves in Figure S13b (Supporting Information), indicate there is almost no fluctuation at a series of potentials ranging from −0.4 to −0.8 V, demonstrating superior durability in the catalytic process. It is worth mentioning from Figure 3a,b that when the applied potential moves lower than −0.5 V, both the NH_3_ yield rates and FEs of catalysts significantly decrease due to the competing HER. To double‐check the production of NH_3_ from CNMS, Nessler's tests and ion chromatography (IC) methods were also employed to determine the production rate of NH_3_. The comparable results obtained by the Nessler's reagent are shown in Figure S14 (Supporting Information), confirming the reliability of the detection results of the indophenol blue method. As shown in Figure S15 (Supporting Information), the Na^+^ peak (in 4.10 min) is so high because of the Na_2_SO_4_ electrolyte, which almost covers the NH_4_^+^ peak (in 4.8 min), resulting in the unsuitability of the IC method in this case. Besides, the quantified nuclear magnetic resonance (NMR) experiments have been operated and are shown in Figure S16 (Supporting Information). One can see that the produced ^14^NH_4_^+^ in the electrochemical reaction over CNMS for 1 h and 2 h by feeding ^14^N_2_ is a diploid relationship, in which the produced ^14^NH_4_^+^ for 2 h is close to that by indophenol blue method (Figure S16d, Supporting Information), further indicating the reliability of the ammonia detection by the indophenol blue method. Thus, the performance of CNMS compares favorably to the NRR behaviors of most reported 1T‐MoS_2_ electrocatalysts operated under ambient conditions, such as 1T‐MoS_2_/g‐C_3_N_4_ (29.97 µg h^–1^ mg^–1^_cat._, 20.48%), 1T‐MoS_2_ @Ti_3_C_2_ (30.33 µg h^–1^ mg^–1^_cat._, 10.94%), 1T‐MoS_2_/BTAB/PPy/GO (7.05 µg h^–1^ mg^–1^_cat._, 5.88%) and many other aqueous‐based NRR electrocatalysts, including Fe‐N/C‐CNTs (34.83 µg h^–1^ mg^–1^_cat._, 9.28%), Pd‐Co/CuO (10.04 µg h^–1^ mg^–1^_cat._, 2.16%),and Au/TiO_2_ (21.40 µg h^–1^ mg^–1^_cat._, 8.11%). A more detailed comparison is summarized in Table S2 (Supporting Information). Moreover, the performance of CNMS was also evaluated in 0.1 м HCl and 0.1 м KOH solution (Figure S17, Supporting Information), and lower NH_3_ yield rates and smaller FEs are obtained in the above electrolytes compared to the neutral Na_2_SO_4_ solution, resulting from the sharply promoted competitive HER.^[^ ^33^, ^36^, ^38^ ^]^

To validate the reaction product indeed originating from the NRR catalyzed by CNMS, the potential nitrogen source contaminant caused by the incomplete dissolution of g‐C_3_N_4_ in CNMS should be clarified. First, we can see from Figure S7d (Supporting Information) that there is no peak at 402.3 eV in all samples,^[^ ^36^ ^]^ demonstrating that no NH_4_^+^ ion exists in the catalysts, but other N species. Second, as shown in Table S1 (Supporting Information), the atomic percent of N element in MoS_2_ obtained from the EDX analysis is 7.5%, and the corresponding catalyst mass loaded on the electrode is about 0.015 mg. Assuming that the N species in the catalysts are completely converted to NH_4_^+^, the NH_3_ yield rate of the potential nitrogen source contaminant should be about 3.98 µg h^–1^ mg^–1^_cat._, which is much lower than that of 71.07 µg h^–1^ mg^–1^_cat._, indicating that the N‐contamination caused by the incomplete dissolution of g‐C_3_N_4_ in CNMS is much lower than N_2_ for the reductive production of ammonia. Moreover, an isotopic labeling experiment was further conducted using ^15^N_2_. And the products were measured by the ^1^H NMR spectroscopy. As shown in Figure 3c, the ^1^H NMR spectra display a doublet coupling for ^15^NH_4_^+^ and a triplet coupling for ^14^NH_4_^+^ with ^15^N_2_ and ^14^N_2_ as feeding gas, corresponding to the spectra of their standard substances, respectively. What's more, almost no NH_3_ was detected when the NRR was conducted at open circuit potential, in an Ar‐saturated 0.1 м Na_2_SO_4_ solution, or using a bare GCE as the working electrode without CNMS (Figure S18, Supporting Information). Figure S19 (Supporting Information), demonstrates bare g‐C_3_N_4_ has almost no catalytic activity for the NRR, eliminating the effect of the incomplete dissolution of g‐C_3_N_4_. For the reaction catalyzed by CNMS at −0.5 V with switching 2 h cycles between N_2_‐ and Ar‐saturated 0.1 м Na_2_SO_4_ solution (Figure 3d), the NH_3_ can only be detected in the electrolysis process of the N_2_‐saturated solution. The results above powerfully indicate that the generated ammonia indeed originates from N_2_ fixation by CNMS. After the electrolysis, N_2_H_4_ is not detected at the optimum potential, as revealed in Figure S20 (Supporting Information), proving that CNMS possesses excellent selectivity toward NH_3_ production.

The stability is also evaluated and the corresponding results are shown in Figure 3e. After 24 h electrolysis, the CNMS still maintains good electrochemical durability with only a slight fluctuation of current density at −0.5 V, as well as both the NH_3_ yield rate and the FE (Figure S21, Supporting Information). Moreover, both NH_3_ yield rates and FEs of CNMS have negligible changes after six times cycling electrolysis, as revealed in Figure 3f. Besides, the linear correlation between the NH_3_ yield and reaction time during the NRR catalytic process is also presented in Figure S22 (Supporting Information), demonstrating a stable production of NH_3_. The XRD patterns (Figure S23, Supporting Information) affirm the MoS_2_ nature without remarkable changes and XPS spectra (Figure S24, Supporting Information) prove that the CNMS presents no obvious change over valence states and crystalline phases after the NRR. Furthermore, the TEM image (Figure S25a, Supporting Information) proves the preservation of nanoflower features, and the HRTEM image (Figure S25b, Supporting Information) reveals that the 1T phase of MoS_2_ is well‐maintained after the long‐term electrolysis. Consequently, it is convincingly exemplified that the CNMS catalyst possesses extraordinarily electrochemical catalytic activity, selectivity and stability for the NRR.

**Figure 4:**
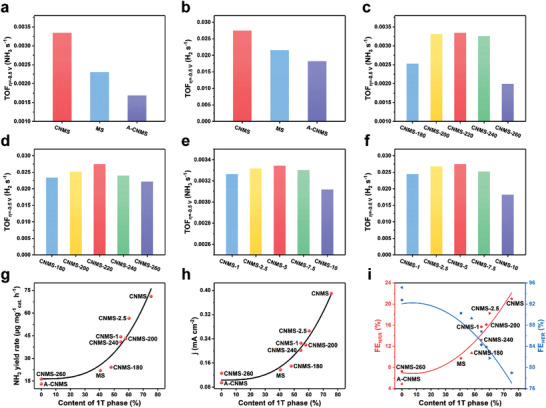
Evolution of a) TOF_NRR_ and b) TOF_HER_ in CNMS, MS, and A‐CNMS. Evolution of c) TOF_NRR_ and d) TOF_HER_ versus hydrothermal temperatures. Evolution of e) TOF_NRR_ and f) TOF_HER_ versus the g‐C_3_N_4_/MoS_2_ ratio. g) Evolution of the NH_3_ yield rates versus the content of 1T phase. h) Evolution of the current density at −0.5 V versus the content of 1T phase. i) Evolution of FEs versus the content of 1T phase, and points of the same shape represent the same sample.

We further explored the improvement mechanism of the superior catalytic performance of CNMS toward the electrocatalytic NRR. The electrochemical active surface area (ECSA) was collected to estimate the number of active sites (Figure S26 and Table S3, Supporting Information). Note that the ECSA of CNMS is 30.2 cm^–2^, much larger than those of MS (13.5 cm^–2^) and A‐CNMS (11.0 cm^–2^), indicating more exposure of active sites. As depicted in Figure S27a (Supporting Information), the charge transfer resistance (*R*_ct_) of the CNMS is much smaller than those of MS and A‐CNMS, which should be attributed to a higher content of metallic 1T phase in CNMS, which would facilitate the electron transfer ability and thus efficiently promote the NRR kinetics. The nitrogen temperature‐programmed desorption (TPD) was conducted to investigate the nitrogen adsorptive capacity. As shown in Figure S27b (Supporting Information), the desorption peaks of CNMS at 200–350 ℃ assigned to the chemical adsorption are much higher than those of MS and A‐CNMS, implying the outstanding nitrogen adsorption ability, consistent with the results of ECSA. Considering the competitive relationship between HER and NRR, HER tests were also conducted in Ar‐saturated 0.1 м Na_2_SO_4_ solution to understand the evolution of the intrinsic activities. We can see from **Figure** **4**a,b and Figure S28 (Supporting Information) that the evolution of TOF_NRR_ and TOF_HER_ are highly consistent with the evolution of 1T contents (Figure S5, Supporting Information), indicating that the presence of 1T phase can accelerate both NRR and HER.

To get an optimized parameter, the influence of preparation temperature was also investigated. Figure S29 (Supporting Information) displays the NRR performances of all the catalysts prepared at various temperatures. One can see that with the increased preparation temperatures, the NRR performances are gradually increased and reach the maximum at 220 ℃ and then decrease at higher temperatures. The ECSA tests (Figure S30 and Table S4, Supporting Information) clearly show that the CNMS‐220 shows a significantly larger ECSA value than other counterparts, providing more active sites. And the CNMS‐260 shows a remarkably decreased ECSA value, which can be ascribed to the absence of 1T phase. In addition, electrochemical impedance spectroscopy (EIS) was also acquired, as exhibited in Figure S31a (Supporting Information). The *R*_ct_ is gradually decreased with the increased temperature and achieves the minimum value over CNMS‐220, and then rises up. Based on the N_2_‐TPD spectra (Figure S31b, Supporting Information), we can see that the CNMS‐220 exhibits the highest signal, indicating the strongest adsorption ability on N_2_ molecules due to the highest content of 1T phase. Moreover, the evolution of the TOF_NRR_ and TOF_HER_ versus the hydrothermal temperatures are depicted in Figure 4c,d and Figure S32 (Supporting Information). It is obvious that both the TOF_NRR_ and TOF_HER_ are enlarged with the increased hydrothermal temperatures and reach maximum at CNMS‐220, and then get down, consistent with the evolution of 1T content versus hydrothermal temperatures (Figure S8, Supporting Information), further demonstrating the leading function of 1T phase in the NRR and HER processes.

Subsequently, we further attempted to figure out the role of the g‐C_3_N_4_/MoS_2_ ratio in NRR. It can be observed from Figure S33 (Supporting Information) that the evolution of NRR properties with increased g‐C_3_N_4_ contents shows a “volcano” trend. Note that the properties of CNMS‐10 are lower than that of CNMS‐7.5, while they have the similar 1T content. This is because excess g‐C_3_N_4_ leads to rigorous aggregations, as revealed in Figure S34 (Supporting Information). Moreover, the cyclic voltammetry curves (CVs) and calculated *C*_dl_ are shown in Figure S35 (Supporting Information) and the corresponding ECSA values are summarized in Table S5 (Supporting Information). One can see that the ECSA of CNMS‐10 is inferior to that of CNMS‐7.5 due to serious aggregations, which is in agreement with SEM images in Figure S34 (Supporting Information). EIS tests (Figure S36a, Supporting Information) display that the CNMS‐5 possesses the minimum *R*_ct_, suggesting more rapid charge transfer due to the highest 1T content. Moreover, when the g‐C_3_N_4_/MoS_2_ ratio achieves 5, the obtained CNMS‐5 shows the highest N_2_ adsorption ability (Figure S36b, Supporting Information). Furthermore, we also conducted the evolution of the TOF_NRR_ and TOF_HER_ versus the g‐C_3_N_4_/MoS_2_ ratio (Figure 4e,f and Figure S37, Supporting Information). Obviously, the evolution of TOF_NRR_ and TOF_HER_ are highly consistent with the evolution of 1T contents versus the g‐C_3_N_4_/MoS_2_ ratio (Figure S10, Supporting Information), further suggesting the dependence of the NRR and HER properties with the 1T content.

In addition, we collected the NH_3_ yield rate at −0.5 V in N_2_‐saturated 0.1 м Na_2_SO_4_ as NRR performance and the current density measured at −0.5 V in Ar‐saturated 0.1 м Na_2_SO_4_ as HER performance of all samples and plotted the evolution of them versus the 1T content, as shown in Figure 4g,h, respectively. It can be seen that both the NH_3_ yield rate and the current density at −0.5 V are positively related to the content of 1T phase, demonstrating that the 1T phase plays an active role in both NRR and HER. Finally, the FE evolution of NRR and HER are plotted in Figure 4i, in which the FEs of NRR are increased with the enlarged 1T content, while the FEs of HER are on the contrary, which suggests that the selectivity toward NRR is also directly proportional to the 1T content, and the performance growth of NRR (Figure 4g) with the increase of 1T content is faster than that of HER (Figure 4h). Hence, it can be concluded that the 1T phase can synchronize the selectivity and activity in the NRR process.

**Figure 5:**
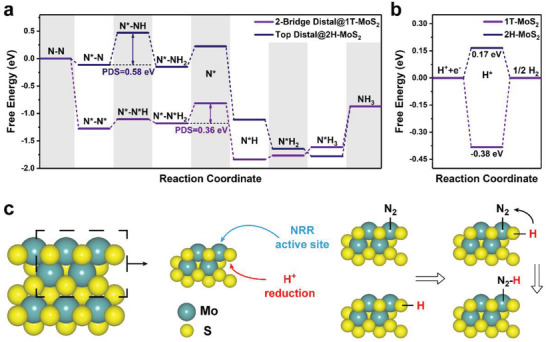
a) Gibbs free energy diagrams on the 1T‐MoS_2_ surface along the respective optimum NRR pathway and the 2H‐MoS_2_ surface. The PDS for each reaction pathway is marked. b) HER Gibbs free energy diagrams on the 1T‐MoS_2_ and 2H‐MoS_2_ surface. The asterisk * in a) and b) denotes the atom in the adsorbate which is bound to the 1T‐MoS_2_ and 2H‐MoS_2_ surface. The common intermediates for all reaction pathways on the 1T‐MoS_2_ and 2H‐MoS_2_ surface are denoted in the black font. c) Initial model of 1T‐MoS_2_ and the hypothesis that NRR is catalyzed by 1T‐MoS_2_ in a synergetic process in which the Mo edge of 1T‐MoS_2_ acts as a proper NRR active site while the basal plane on pristine behaves as an HER active site. Once N_2_ is adsorbed, a reduced proton on the edge of the basal plane will subsequently provide hydrogen to hydrogenate the N_2_.

First principle calculations based on density functional theory (DFT) were performed to figure out the likely mechanism of the overall NRR on the combined entity of 1T and 2H phases of MoS_2_. Figures S38–S42 (Supporting Information) summarize the optimized atomic structures along several feasible NRR pathways as well as others considered. The optimal one was chosen to compare with 2H‐MoS_2_ in consideration of both N_2_ adsorption energy and the potential‐determining step (PDS), and the obtained Gibbs free energy diagrams are illustrated in **Figure** **5**a. One can see that the 1T‐MoS_2_ is able to absorb the inert N_2_ more remarkably than the 2H phase at Mo‐edge with $\Delta G_{1T-MoS_{2}}$ (N—N→N*—N*) = −1.28 eV and $\Delta G_{2H-MoS_{2}}$ (N—N→N*—N) = −0.11 eV, respectively. The PDS is the hybrid step containing the hydrogenation of N*—N*H_2_ to generate N*—NH_3_ and the desorption of the first NH_3_ for 1T‐MoS_2_ ($U_{\mathrm{limiting}-1T-\mathrm{Mo}S_{2}}^{\mathrm{NRR}}=\;-0.36\;V$), while it is only the first hydrogenation of N*—N to produce N*—NH for 2H‐MoS_2_ ($U_{\mathrm{limiting}-2H-\mathrm{Mo}S_{2}}^{\mathrm{NRR}}=-0.58\;V$). Furthermore, from the charge density difference (Figure S43, Supporting Information), it can be clearly seen that more electrons tend to accumulate around the edge Mo atoms of 1T phase than that of 2H phase, indicating the change of electronic structure and more intensive N_2_ adsorption and activation ability, which is in coincidence with the XPS (Figure 2c) and TPD (Figure S27b, Supporting Information) results. All the results above suggest that the Mo‐edge of 1T phase is more catalytically active toward NRR than that of 2H phase.

It is well known that the HER is the major competing reaction against NRR. Hence, the HER activities on the Mo‐edge of 1T and 2H phases were also investigated, as exhibited in Figure 5b. Analogous to NRR, the hydrogen atom can be bound to the Mo‐edge of 1T phase with the hydrogen desorption/combination as the PDS ($U_{\mathrm{limiting}-1T-\mathrm{Mo}S_{2}}^{\mathrm{HER}}=\;-0.38\;V$). According to previous studies,^[^ ^45^, ^46^, ^47^ ^]^ the selectivity of an electrocatalyst between NRR and HER can be qualitatively judged by comparing the change of Gibbs free energy for the first step (NRR: N_2_ → N*_2_; HER: H^+^ + e^–^ → H*) and the PDS. When an electrode potential of $U_{\mathrm{limiting}-1T-\mathrm{Mo}S_{2}}^{\mathrm{NRR}}$ is applied, Δ*G* (N_2_ → N*_2_) = −1.28 eV is still more negative than Δ*G* (H^+^ + e^–^ → H*) = −0.74 eV, as well as Δ*G* (H_2_O → H_2_O*) = −0.03 eV (Figure S44, Supporting Information), suggesting that the Mo‐edges of 1T phase are covered by the nitrogen, rather than hydrogen and water adsorbates. Besides, considering the limiting potential, $|U_{\mathrm{limiting}-1T-\mathrm{Mo}S_{2}}^{\mathrm{NRR}}\left|<\right|U_{\mathrm{limiting}-1T-\mathrm{Mo}S_{2}}^{\mathrm{HER}}|$ implies that on the Mo‐edges of 1T phase the barrier for hydrogen desorption/combination cannot be overcome at $U_{\mathrm{limiting}-1T-\mathrm{Mo}S_{2}}^{\mathrm{NRR}}$. Therefore, the Mo‐edge of 1T phase favors the NRR. As for 2H‐MoS_2_, when an electrode potential of $U_{\mathrm{limiting}-2H-\mathrm{Mo}S_{2}}^{\mathrm{NRR}}$ is applied, Δ*G* (H^+^ + e^–^ → H*) = −0.41 eV is negative than Δ*G* (N_2_ → N*_2_) = −0.11 eV, indicating that the Mo‐edge of 2H phase tends to be covered by hydrogen adsorbates. Besides, $|U_{\mathrm{limiting}-2H-\mathrm{Mo}S_{2}}^{\mathrm{HER}}\left|<\;\right|U_{\mathrm{limiting}-2H-\mathrm{Mo}S_{2}}^{\mathrm{NRR}}|$ suggests that the Mo‐edge of 2H‐MoS_2_ prefers the HER.

Generally, the basal plane of 1T‐MoS_2_ is also considered as active sites for the HER.^[^ ^36^, ^38^ ^]^ In the basal plane, the calculated free energy of hydrogen adsorption (${\Delta G}_{\mathrm{basal}\;\mathrm{plane}}^{H}$) as a function of surface H coverage in the absence of water and a bias potential is shown in Figure S45 (Supporting Information). The adsorption geometries at different H coverages are provided in Figure S46 (Supporting Information). At a low H coverage (<25%) in the initiation of catalysis, the ${\Delta G}_{\mathrm{basal}\;\mathrm{plane}}^{H}$ value (−1.90 eV) is more negative than that of ${\Delta G}_{\mathrm{Mo}-\mathrm{edge}}^{H}$ (−0.38 eV), indicating the adsorbed H prefers binding to S atoms in the basal plane, thus making the N_2_ adsorption easier on the Mo‐edge. In addition, when an electrode potential of $U_{\mathrm{limiting}-1T-\mathrm{Mo}S_{2}}^{\mathrm{NRR}}$ is applied, the barrier for hydrogen desorption/combination on the edge S atoms of the basal plane cannot be overcome, thus the HER will not happen and the adsorbed H will remain on the edge S atoms of the basal plane. In this case, the hydrogenated S atoms can provide a fundamental role in the NRR mechanism as a H‐provider because the formed *H can be transferred directly to nearby bound N_2_ or to the nitrogen reduction intermediates, significantly accelerating the NRR process, as shown in Figure 5c, which is similar to previous studies.^[^ ^48^, ^49^, ^50^ ^]^ At a high H coverage, we can see from Figure S45 (Supporting Information) that the free energy becomes close to zero (the ${\Delta G}_{\mathrm{basal}\;\mathrm{plane}}^{H}$ value is between −0.02 and 0.27 eV around 37.5–50% H coverage). Therefore, it is most likely that the HER process occurs at the later stage of H coverage on the basal plane of 1T‐MoS_2_. Separation of the active sites can partially result in the separation of the space occupied by the adsorbed species. Though the 1T phase can accelerate both the NRR and HER, to a certain degree, it will reduce the competition between HER and NRR due to the active sites separation, thus synchronizing the selectivity and activity for the NRR, as exhibited in Figure 4g,i. Evidently, the site separation analysis based on the DFT calculations is in good agreement with the previous experimental results.

## 3 Conclusions

With the addition of g‐C_3_N_4_, the hybrid 1T/2H MoS_2_ with a high 1T phase content (75.44%) has been successfully produced by a facile hydrothermal route and verified as a highly active and selective NRR electrocatalyst. The optimized CNMS catalyst exhibits a high NH_3_ yield rate of 71.07 µg h^–1^ mg^–1^_cat._ and a large FE of 21.01% at −0.5 V versus RHE in 0.1 м Na_2_SO_4_. The 1T phase of MoS_2_ plays several vital roles: 1) serving as active sites for the NRR. 2) Regulating electronic structure, further accelerating the N_2_ adsorption. 3) Promoting the interfacial charge transfer from catalysts to N_2_ molecules with a smaller resistance. DFT calculations suggest that the enhanced NRR performance of the Mo‐edge in 1T‐MoS_2_ compared with 2H‐MoS_2_ should be rationally associated with a lower energy barrier of PDS, as well as more profitable adsorption of N_2_ molecules due to the presence of 1T phase. Meanwhile, the separation of active sites over 1T phase can reduce the competition between HER and NRR, which contributes to higher Faradaic efficiency toward the NRR, thus achieving the synchronization of selectivity and activity. The present study demonstrates a new strategy on the active sites separation to synchronize the selectivity and activity over 1T phase of MoS_2_ for the multistep catalytic reactions.

## Conflict of Interest

The authors declare no conflict of interest.

## Supporting information

Supporting Information

## Data Availability

Research data are not shared.
