# Supplementary material for: Highly Efficient Electrocatalytic N2 Reduction to Ammonia over Metallic 1T Phase of MoS2 Enabled by Active Sites Separation Mechanism
Source: Adv Sci (Weinh). 2021 Nov 5;9(2):2103583. doi: 10.1002/advs.202103583 (PMC8805567; doi:10.1002/advs.202103583)
Supplement: Supplementary file 1 — Supporting Information [file ADVS-9-2103583-s001.pdf]

© 2021 Wiley-VCH GmbH

ADVANCED SCIENCE Open Access

Supporting Information

for *Adv. Sci.,* DOI: 10.1002/advs.202103583

Highly Efficient Electrocatalytic N2 Reduction to Ammonia over Metallic 1T Phase of MoS2 Enabled by Active Sites Separation Mechanism

Ruoqi Liu[a], *Ting Guo*[a], *Hao Fei*[a], *Zhuangzhi Wu*[a*], *Dezhi Wang*[a*], *Fangyang Liu*[b*]

Supporting Information

for

Highly Efficient Electrocatalytic N_2 Reduction to Ammonia over Metallic 1T Phase of MoS_2 Enabled by Active Sites Separation Mechanism

Ruoqi Liu[a], Ting Guo[a], Hao Fei[a], Zhuangzhi Wu[a*], Dezhi Wang[a*], Fangyang Liu[b*]

[a] School of Materials Science and Engineering, Central South University, Changsha 410083, China

[b] School of Metallurgy and Environment, Central South University, Changsha 410083, China

[*]Correspondence: Z Wu, zwu@csu.edu.cn; D. Wang, dzwang@csu.edu.cn; F Liu, liufangyang@csu.edu.cn

Experimental

1. Chemicals and materials

Thioacetamide (CH_3CH_2NS) and phenol were obtained from the Xilong company. Nafion solution (5 wt%), sodium hydroxide (NaOH), sodium hypochlorite (NaClO), sodium nitroferricyanide (III)($C_5FeN_6Na_2O$), ammonium chloride (NH_4Cl) and sodium sulfate (Na_2SO_4) were purchased from Sigma-Aldrich Chemical Reagent Co., Ltd. Dicyandiamide ($C_2H_4N_4$), Deuterium dimethyl sulfoxide (d6-DMSO), molybdenum pentachloride ($MoCl_5$), nitric acid, sulfuric acid, hydrochloric acid and ethanol were bought from Aladdin Ltd. High purity nitrogen (N_2, ≥99.999 %) and argon (Ar, ≥99.999 %) were provided by Saizhong Special Gas Co., Ltd in Changsha. All reagents were of analytical grade and used without further purification. A Nafion proton exchange membrane (PEM) was purchased from the DuPont Company. The ultrapure water used throughout all experiments was purified through an ultrapure (UP) system.

2. Preparation of g-C_3N_4 nanosheets

The g-C_3N_4 nanosheets were prepared based on previous work:[1] 2 g dicyandiamide powder and 10 g ammonium chloride were fully mixed and then kept at 550 °C for 4 h.

3. Preparation of 1T-MoS_2

Firstly, 0.594 g $MoCl_5$ was dissolved into 20 mL absolute ethanol, and then 1 g g-C_3N_4 were added into the solution under stirring for 10 min. After being stirred to form a transparent solution, the mixture was stirred at 100 °C to evaporate ethanol thoroughly, and the final products were ground into powder. The obtained mixture and 0.52 g thioacetamide were dissolved in 40 mL distilled water under vigorous stirring to form a homogeneous. After being stirred for 1 h, the solution was transferred into a 100 mL Teflon-lined stainless-steel autoclave and maintained at 220 °C for 13 h. After being cooled down, the black product was washed and freeze-dried overnight. The obtained MoS_2 with g-C_3N_4 as a template was named CNMS. For comparison, MoS_2 without using the g-C_3N_4 template was also synthesized under

the same conditions and named MS. Other comparative experiments were also conducted at 180, 200, 220, 240, and 260 ℃ for 13 h, respectively, and the as-prepared samples were correspondingly labeled as CNMS-180, CNMS-200, CNMS-220, CNMS-240, and CNMS-260. Besides, the samples with various MoS_2/g-C_3N_4 ratio were also synthesized following the same process with various contents of g-C_3N_4 (2.970, 1.188, 0.594, 0.396, and 0.297 g), named as CNMS-1, CNMS-2.5, CNMS-5, CNMS-7.5, and CNMS-10.

4. Preparation of working electrodes

The catalyst powder (3 mg) was dispersed in a mixture of 720 μL water, 200 μL ethanol and 80 μL of 5 wt% Nafion, and then sonicated for 30 min. Next, 5 μL of the catalyst ink was introduced dropwise onto a well-polished GCE surface (d = 3 mm) and dried at room temperature.

5. Characterization.

The X-ray diffraction (XRD) patterns were measured using a D/max-2500 X-ray Diffractometer with a Cu Kα radiation (λ= 0.154 nm). The scanning electron microscopy (SEM) images were collected on a FEI Sirion 200 scanning electron microscope. The transmission electron microscopy (TEM) images and high-resolution TEM (HRTEM) images were performed on a JEOL-2100F transmission electron microscope. The X-ray photoelectron spectroscopy (XPS) measurements were conducted on a ESCALAB 250 Xi using Al Kα as the exciting source. The binding energy value of each element was calibrated with C 1s at 284.6 eV. The Raman spectra were acquired using the LabRAMHR-800 (HORIBA, French) at 633 nm.

6. Electrochemical measurements

Before nitrogen reduction reaction (NRR) tests, the Nafion 211 membrane was protonated by heating in 5 % H_2O_2 solution and ultrapure water at 80 °C for 1 h, respectively. The electrochemical experiments were performed with a CHI 660E electrochemical analyzer (CHI Instruments, Inc., Shanghai) using a three-electrode configuration with the modified GCEs by various catalysts as working electrodes, platinum sheet as the counter electrode and

Ag/AgCl electrode (saturated KCl) as the reference electrode. Potentials reported in this work were converted to RHE via calibration with the following equation: E (*vs.* RHE) = E (*vs.* Ag/AgCl) + 0.197 + 0.059 × pH and the presented current densities were normalized to the geometric surface area. Linear sweep voltammetry (LSV) test was conducted with a scan rate of 2 mV s^{-1} in an Ar- and N_2-saturated 0.1 M Na_2SO_4 solution at room temperature. The electrochemical impedance spectroscopy (EIS) was also tested in the frequency range from 1 Hz to 10^6 Hz in an N_2-saturated 0.1 M Na_2SO_4 solution. Each collected aliquot was titrated twice by the indophenol blue method to measure the NH_3 concentration, thus the Faradic efficiency (FE) and their yield rate. For the NRR, electrochemical tests were conducted in the N_2-saturated 0.1 M Na_2SO_4 solution, which was bubbled with N_2 for 30 min before the measurement.

We then executed a series of strict control experiments to eliminate the ammonia contamination and ensure the accuracy of the results. Firstly, the as-prepared catalysts were washed in 0.1 M HCl to remove the NH_4^+ caused by the incomplete dissolution of g-C_3N_4. Secondly, during the bubble process, the feeding gas was pre-purified by continuously passing into the 0.05 M H_2SO_4 to remove the tiny NO_x species in the feeding gas.[2-4] Besides, to remove the contribution of the N-contamination on the NRR performance, four extra control experiments were also performed: (1) CNMS in a N_2-saturated 0.1 M Na_2SO_4 solution at an open circuit potential for 2 h; (2) CNMS in an Ar-saturated 0.1 M Na_2SO_4 solution at -0.5 V for 2 h; (3) a bare GCE in a N_2-saturated 0.1 M Na_2SO_4 solution at -0.5 V for 2 h; (4) CNMS at -0.5 V with alternating 2 h cycles between N_2- and Ar- saturated 0.1 M Na_2SO_4 solution for a total of 12 h.

7. Indophenol blue method

Concentration of produced ammonia was estimated by the indophenol blue method through the ultraviolet spectrum. In details, 6.5 mL of 6 M NaOH solution, 3 mL of 1 wt % sodium nitroprusside solution, and 5 mL of 6.05 M phenol were added into 50 mL of 1.5 M sodium citrate solution. After being stirred for 10 min at room temperature, the indophenol blue solution was obtained and preserved in the refrigerator. To calibrate the concentration-absorbance curve, 10 mL of the standard ammonia chloride solution with

various concentrations of 0, 0.2, 0.4, 0.5, 0.8, and 1.0 μg mL^{-1} were mixed with 1 mL of indophenol blue solution and 1 mL of 0.05 M NaClO. After being stirred for 30 min, 2 mL of the above solution was drawn out for the UV-vis absorbance measurement at 637 nm. We then plotted the profile of absorbance value *vs* the concentrations of standard solution, and the fitting curve (Y = 0.2944X+0.0082, R^2 = 0.999) showed a good linear relation of the absorbance value with the NH_4Cl concentration by thrice independent calibrations. To quantify the generated NH_3, 1 mL of indophenol blue solution and 1 mL of 0.05 M NaClO were added into 10 mL of the reaction solution. Then, 2 mL of the above solution was drawn out for the UV-vis absorbance measurement at 637 nm. According to the light absorbance and standard curve, the ammonia concentration was obtained.

8. Nessler's reagent method

To calibrate the concentration-absorbance curve, 10 mL standard ammonia chloride solution with the concentrations of 0, 0.2, 0.4, 0.5, 0.8, and 1.0 μg mL^{-1} were mixed with 0.5 mL of potassium sodium tartrate solution (0.5 g mL^{-1}) and 0.5 mL of Nessler's reagent solution, respectively. After 30 min, 2 mL of the above solution was drawn out for UV-vis absorbance measurement at 420 nm. We then plotted the profile of absorbance value *vs* the concentrations of standard solution by thrice independent calibrations, where a linear correlation with R^2 = 0.9995 was obtained (Figure S14). To quality the generated NH_3 during electrocatalysis, 0.5 mL of potassium sodium tartrate solution (0.5 g mL^{-1}) and 0.5 mL of Nessler's reagent solution were added into 10 mL of the reaction solution. After 30 min, 2 mL of the above solution was drawn out for UV-vis absorbance measurement at 420 nm. According to the light absorbance and standard curve, the ammonia concentration was obtained.

9. Ion chromatograph method

During detection of NH_4^+, 4.5 mmol L^{-1} of methane sulfonic acid was used as the eluent solution with a flow rate of 1 mL min^{-1}. The column temperature and self-regenerating suppressor (SRS) current were kept as 35 °C and 30 mA, respectively. To calibrate the concentration-area curve, 1 mL cationic composition analysis standard solution with

concentrations of 0, 0.2, 0.5, 0.8, 1.0, and 2.0 μg mL^{-1} were injected through the 50 μL quantitative injection loop to achieve standard analyte peaks for low concentrations of NH_4^+, and the retention time of NH_4^+ was about 4.78 min. By twice independent calibrations, we plotted the profile of peak areas *vs* the concentrations of standard solution, where a linear correlation with R^2=0.999 was obtained (Figure S15). To quality the generated NH_3 during electrocatalysis, the same volume of reaction solution was analyzed using the same method. According to the peak areas and standard curve, the concentration of the generated ammonia was obtained. Noting that the acid in eluent solution guarantees the generated NH_3 transformed to NH_4^+ to be detected by the ion chromatograph.

10. Determination of N_2H_4

The N_2H_4 in the electrolyte was estimated by the method of Watt and Chrisp. The p-$C_9H_{11}NO$ (5.99 g), 0.1 M Na_2SO_4 (30 mL), and C_2H_5OH (300 mL) were mixed and used as the color reagent. In a typical procedure, 5 mL of electrolyte was removed from the electrochemical reaction vessel and added into 5 mL of above prepared color reagent, and then stirred for 15 min at 25 °C. Besides, the obtained calibration curve of N_2H_4 is Y = 0.1878X + 0.0059 (R^2 = 0.999) by 3 times independent calibrations.

11. ^{15}N isotope labeling experiments

To further verify the origin of generated ammonia, ^{15}N isotope labeling experiments were performed. Considering the limited supply and expensive cost of $^{15}N_2$ gas, 2 bars of $^{15}N_2$ was charged to the cathode chamber instead of bubbling $^{15}N_2$ for 30 min. Noting that before charging $^{15}N_2$, the cathode chamber was charged with Ar for 30 min to remove impurity gas. The product was analyzed by ^1H NMR spectra. For ^1H NMR measurement, the *pH* value of reaction solution was adjusted to 2 by sulfuric acid, which was then concentrated to about 100 μL. Finally, 0.6 mL of d6-DMSO was added into the solution followed by measurement of ^1H NMR spectroscopy.

12. Calculations of NH_3 formation rate and FE

The FE for the N_2 reduction was defined as the amount of electric charge which was

used for synthesizing NH_3 divided the total charge passed through the electrodes during the electrolysis. The total amount of NH_3 was measured using colorimetric methods. Assuming three electrons were needed to produce one NH_3 molecule, the FE could be calculated as follows:

$$\mathrm{FE}=\frac{3\times F\times [NH_4^+]\times V}{17\times Q} \qquad \text{Equation (1)}$$

The rate of NH_3 formation was calculated using the following equation:

$$\text{Ammonia formation rate}=\frac{[NH_4^+]\times V}{m\times t} \qquad \text{Equation (2)}$$

Where F is the Faraday constant (96485.3 C mol^{-1}), $[NH_4^+]$ is the measured NH_3 concentration, V is the volume of the cathodic reaction electrolyte, Q is the quantity of applied electricity, t is the reduction time and m is the catalyst mass.

13. TOF calculation

To calculate turnover frequency (TOF) in the HER and NRR processes for each site, we used the following formula:[5-8]

$$\mathrm{TOF}_{(\mathrm{HER/NRR})}=\frac{\text{number of total product turnovers/cm}^2\text{ of geometric area}}{\text{number of active sites/cm}^2\text{ of geometric area}} \qquad \text{Equation (3)}$$

Calculate the total number of product (H_2 or NH_3) turnovers according to the current density:

$$\text{no. of } \mathrm{H_2}=\left(j_{HER}\frac{\mathrm{mA}}{\mathrm{cm}^2}\right)\left(\frac{1\ \mathrm{C\ s^{-1}}}{1000\ \mathrm{mA}}\right)\left(\frac{1\ \mathrm{mol\ e^{-1}}}{96485.3\ \mathrm{C}}\right)\left(\frac{1\ \mathrm{mol\ H_2}}{2\ \mathrm{mol\ e^{-1}}}\right)\left(\frac{6.022\ \mathrm{mol}\times10^{23}\ \mathrm{H_2\ moleculars}}{1\ \mathrm{mol\ H_2}}\right)$$

$$=3.12\times10^{15}\,\frac{\mathrm{H_2/s}}{\mathrm{cm}^2}\text{ per }\frac{\mathrm{mA}}{\mathrm{cm}^2} \qquad \text{Equation (4)}$$

$$\text{no. of } \mathrm{NH_3}=\left(j_{NRR}\frac{\mathrm{mA}}{\mathrm{cm}^2}\right)\left(\frac{1\ \mathrm{C\ s^{-1}}}{1000\ \mathrm{mA}}\right)\left(\frac{1\ \mathrm{mol\ e^{-1}}}{96485.3\ \mathrm{C}}\right)\left(\frac{1\ \mathrm{mol\ NH_3}}{3\ \mathrm{mol\ e^{-1}}}\right)\left(\frac{6.022\ \mathrm{mol}\times10^{23}\ \mathrm{NH_3\ moleculars}}{1\ \mathrm{mol\ NH_3}}\right)$$

$$=2.08\times10^{15}\,\frac{\mathrm{NH_3/s}}{\mathrm{cm}^2}\text{ per }\frac{\mathrm{mA}}{\mathrm{cm}^2} \qquad \text{Equation (5)}$$

Because the surface Mo ions of MoS_2 were considered to be the catalytic active species. On the basis of electrochemical active surface areas together with the unit cell (one Mo atom and two S atoms with volume of 53.1 $Å^3$) of the MoS_2 crystal structure in the cases of CNMS, similar methods were used to calculate TOF for them. Active site per actual surface area:

$$\text{Active sites}_{MoS_2}=\left(\frac{3\ \text{atom/unit cell}}{53.1\ Å^3\text{/unit cell}}\right)^{2/3}=1.47\times10^{15}\ \text{atoms cm}^{-2} \qquad \text{Equation (6)}$$

According to the above results, the TOF can be calculated as follows:

$$\text{TOF}_{\text{HER}}=\frac{3.12\times10^{15}\,\frac{H_2/s}{cm^2}\ \text{per}\ \frac{mA}{cm^2}}{1.47\times10^{15}\ \text{atoms cm}^{-2}\times A_{ECSA}}\times|j_{HER}| \qquad \text{Equation (7)}$$

$$\text{TOF}_{\text{NRR}}=\frac{2.08\times10^{15}\,\frac{H_2/s}{cm^2}\ \text{per}\ \frac{mA}{cm^2}}{1.47\times10^{15}\ \text{atoms cm}^{-2}\times A_{ECSA}}\times|j_{NRR}| \qquad \text{Equation (8)}$$

14. Computational Details

The present first principle calculations are performed using Vienna ab initio Simulation Package (VASP) with the projector augmented wave (PAW) method based on density functional theory (DFT).[9] The exchange-functional is treated using the generalized gradient approximation (GGA) of Perdew-Burke-Ernzerhof (PBE) functional.[10, 11] The cut-off energy of the plane-wave basis was set at 500 eV for the calculations of atoms optimization. The electronic and ionic convergence criteria were set to be 1×10^{-6} eV and 0.01 eV/Å, respectively.

The monolayer MoS_2 was selected in this work. For Mo edge site, a periodic 3×3×1 supercell with 4×1×1 Γ-centered Monkhorst-Pack k-mesh was used to conduct the surface analysis and calculation. For S basal plane site, a periodic 4×4×1 supercell with 2×2×1 Γ-centered Monkhorst-Pack k-mesh was used. For both surface models, a vacuum layer of around 15 Å was added in the direction perpendicular to the surface to eliminate the spurious interlayer interaction.

The Gibbs free energy is calculated using the following equation:

$\Delta G = \Delta E + \Delta E_{ZPE} - T\Delta S$

where ΔE is the reaction energy between the products and the reactants of the elementary step, which is corrected by the change in the zero-point energy (ΔE_{ZPE}) and the vibrational

entropy (*ΔS*) at *T* = 298.15 K.

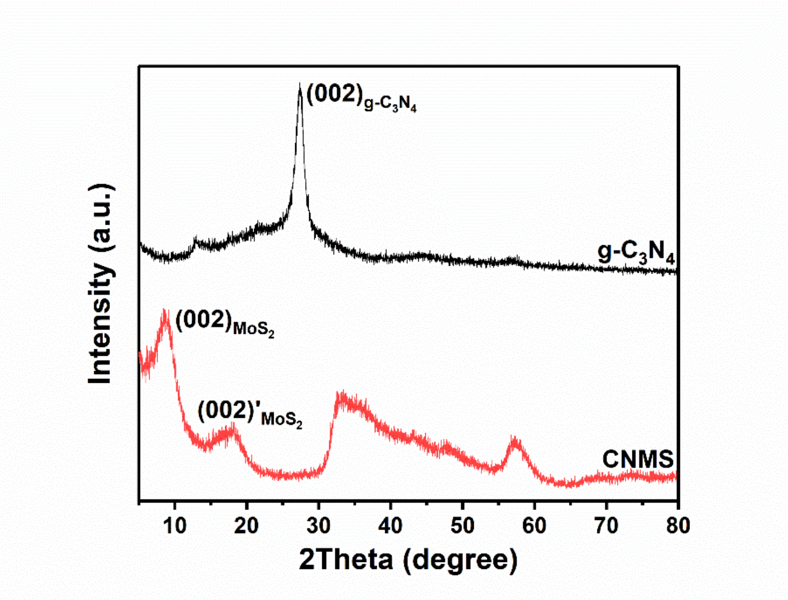

Figure S1. XRD pattern of g-C_3N_4 and CNMS.

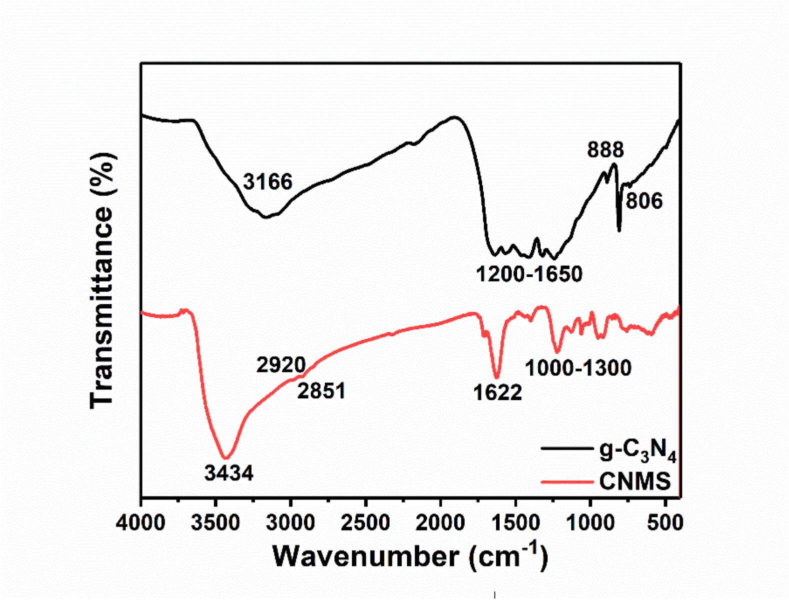

Figure S2. FTIR spectra of g-C_3N_4 and CNMS.

The absorption peak of g-C_3N_4 at 3166 cm^{-1} could be related to the N-H stretching vibration.[12] Besides, the characteristic peak in the range of 1200-1650 cm^{-1} is associated with the stretching vibration of CN heterocycles,[13] and the peak at 806 and 888 cm^{-1} are ascribed to the breathing mode of the triazine ring.[14] As for CNMS, it is obvious that the peaks of triazine ring have disappeared, indicating the decomposition of the ring structure, while some small carbonic molecules appear at the same time. The characteristic peaks at 3434 cm^{-1} and 1622 cm^{-1} are assigned to the stretching vibration and bending vibration of the hydroxyl group in the surface water molecular.[14] The peaks formed at 2920 and 2852 cm^{-1} correspond to the -CH_2 groups.[15] According to the work of Lartin,[16, 17] C-C and C-O bonds have extremely similar force constants, so the region of 1000-1300 cm^{-1} can be related to C-C and C-O stretching vibration.

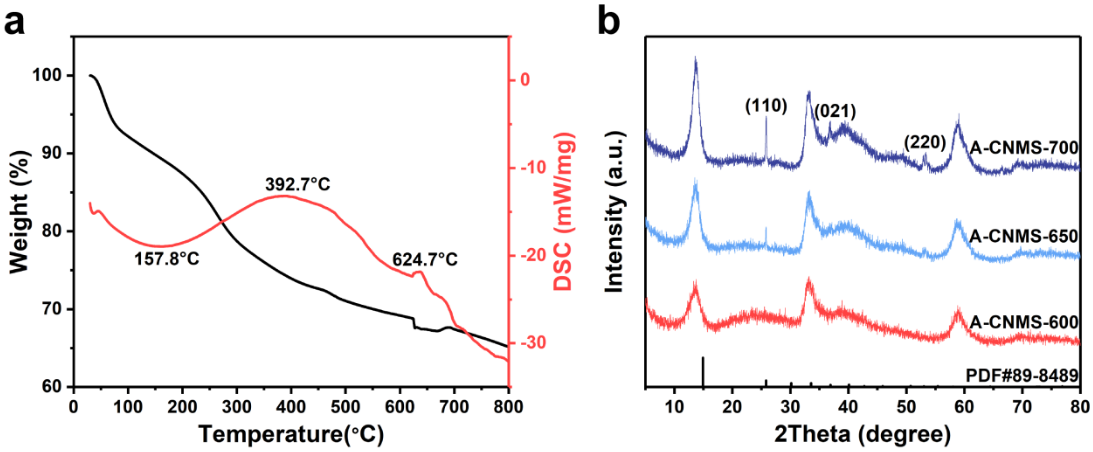

Figure S3. (a) TG/DSC curves of CNMS. (b) XRD patterns of annealed A-CNMS at 600, 650, and 700 °C.

DSC curves of CNMS (Figure S3a) present an obvious endothermic peak at 157.8 °C, which is ascribed to the vaporization water molecular absorbed on the surface of MoS_2.[18] Moreover, there is an exothermic peak at 392.7 °C, corresponding to a structural conversion from the metastable 1T phase to the stable 2H phase of MoS_2, which is greatly higher than the transformation temperature (95 °C) of the monolayer 1T,[19] implying that the intercalation materials contribute to stabilizing 1T-MoS_2. Another exothermic peak appears at 624.7 °C. To investigate the evolution of samples in this temperature range, calcination of CNMS at 600, 650, and 700 °C was conducted and the XRD pattern is shown in Figure S3b. No typical graphite characteristic peaks are detected at the annealing temperature of 600 °C. When the calcination temperature reaches 650 °C, the characteristic peaks of graphite appear, and the intensity is increased at a higher annealing temperature of 700 °C. The analysis above indicates this exothermic peak is attributed to the graphitization of small carbonic molecules.

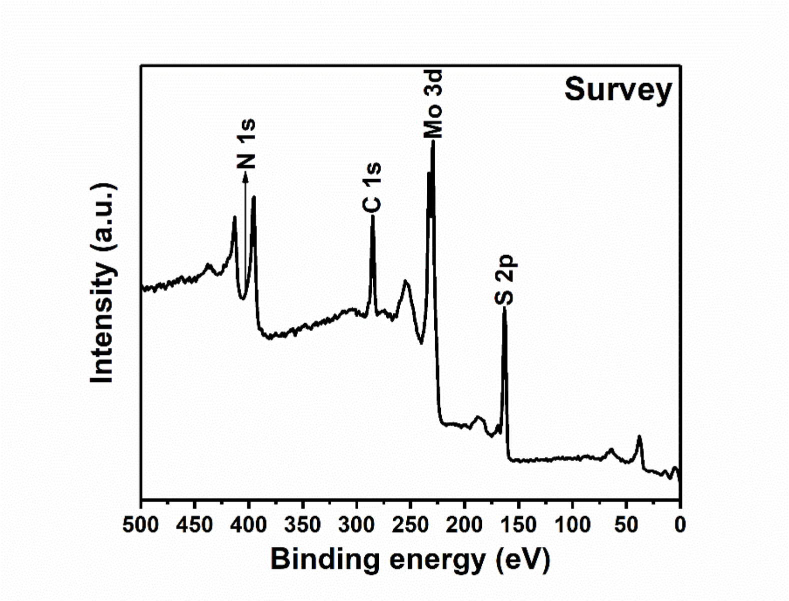

Figure S4. The XPS survey of CNMS.

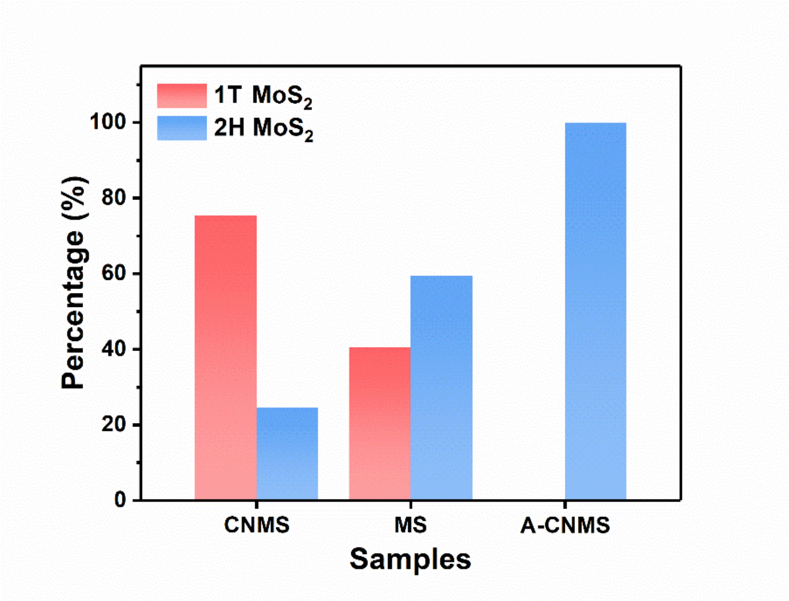

Figure S5. Relative fractions of 1T and 2H phases of CNMS, MS, and A-CNMS.

Table S1. Element contents of CNMS from XPS and EDX.

Element	Mo	S	C	N	C/N
XPS	19.2 at. %	35.9 at. %	41.5 at. %	3.4 at. %	12.2
EDX	16.9 at. %	34.9 at. %	40.7 at. %	7.5 at. %	5.4

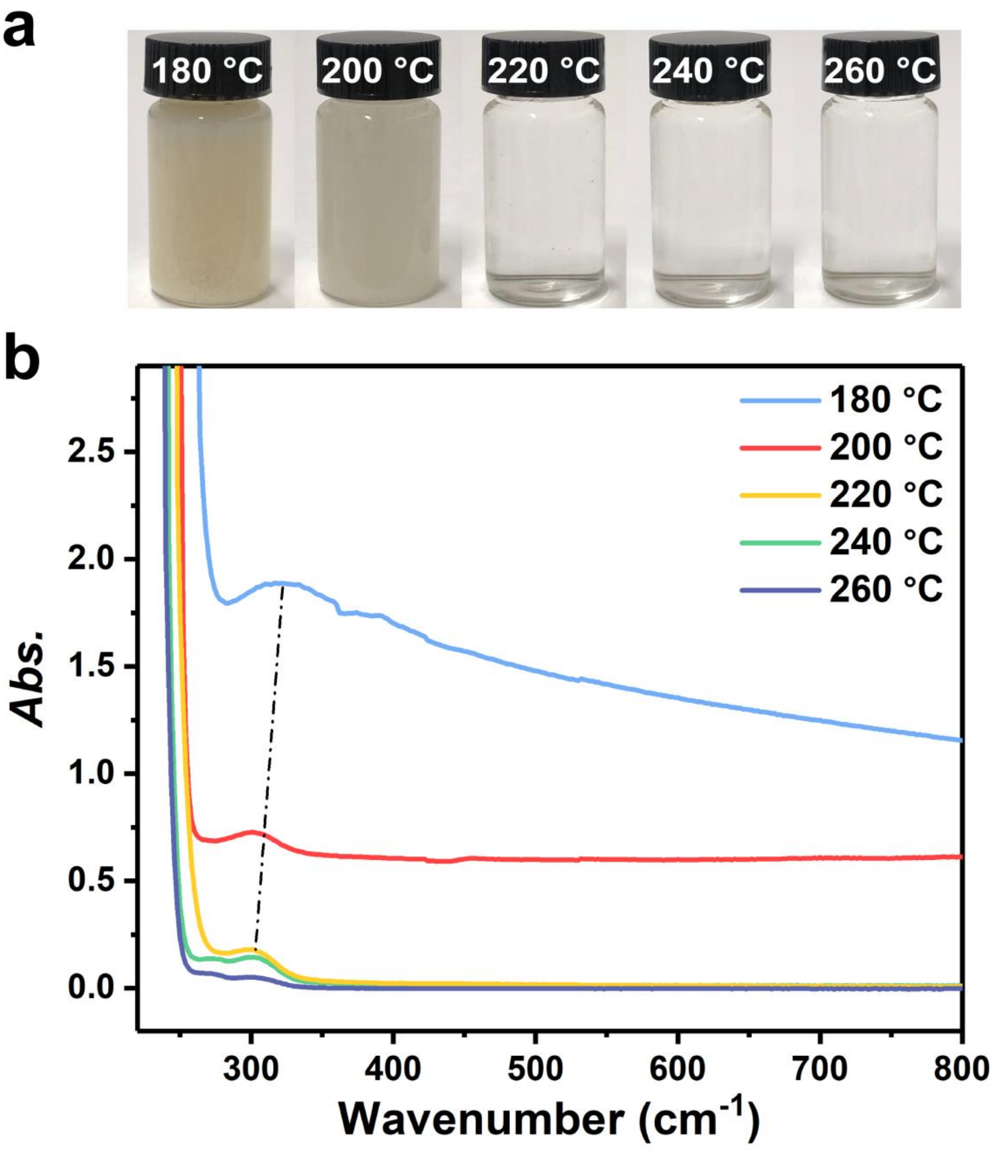

Figure S6. (a) Digital photos of g-C_3N_4 suspension after hydrothermal treatments at 180, 200, 220, 240, and 260 °C. (b) UV-Visible absorption spectra of the corresponding g-C_3N_4 samples.

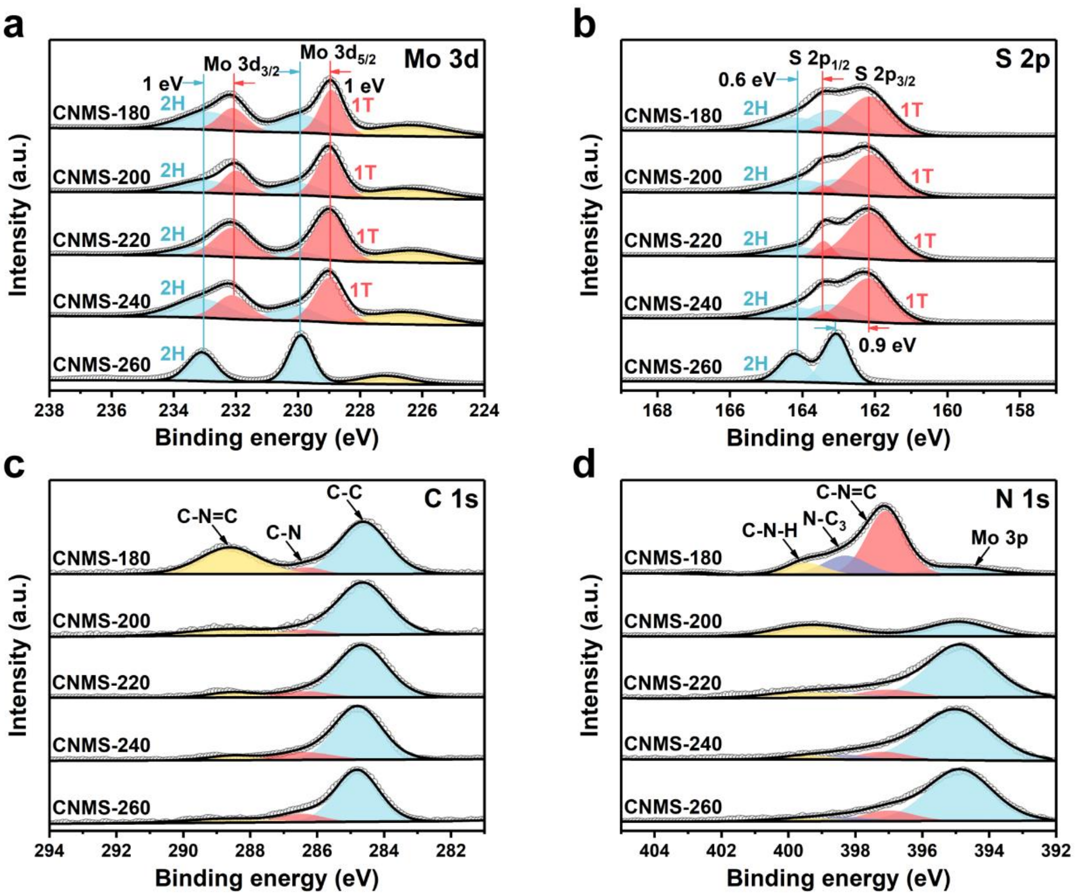

Figure S7. XPS spectra of (a) Mo 3d, (b) S 2p, (c) C 1s, and (d) N 1s of CNMS-180, CNMS-200, CNMS-220, CNMS-240, and CNMS-260.

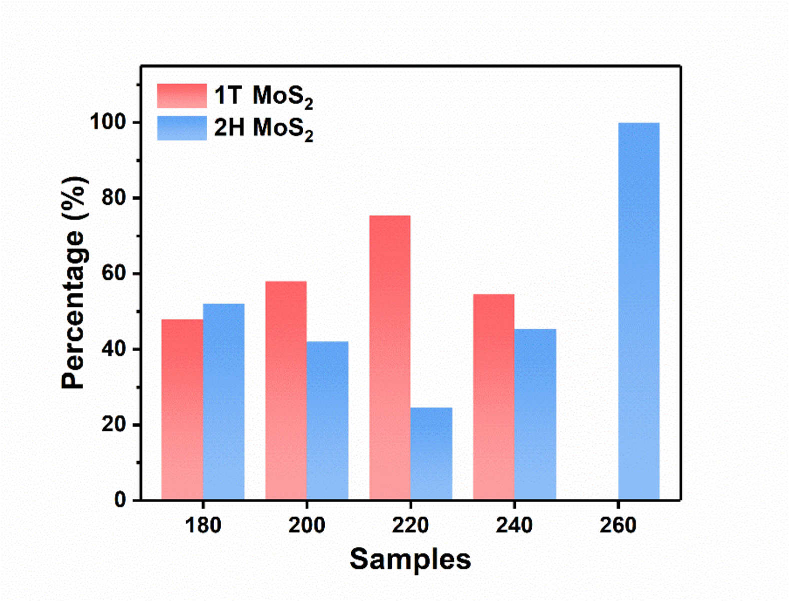

Figure S8. Relative fractions of 1T and 2H phases of CNMS-180, CNMS-200, CNMS-220, CNMS-240, and CNMS-260.

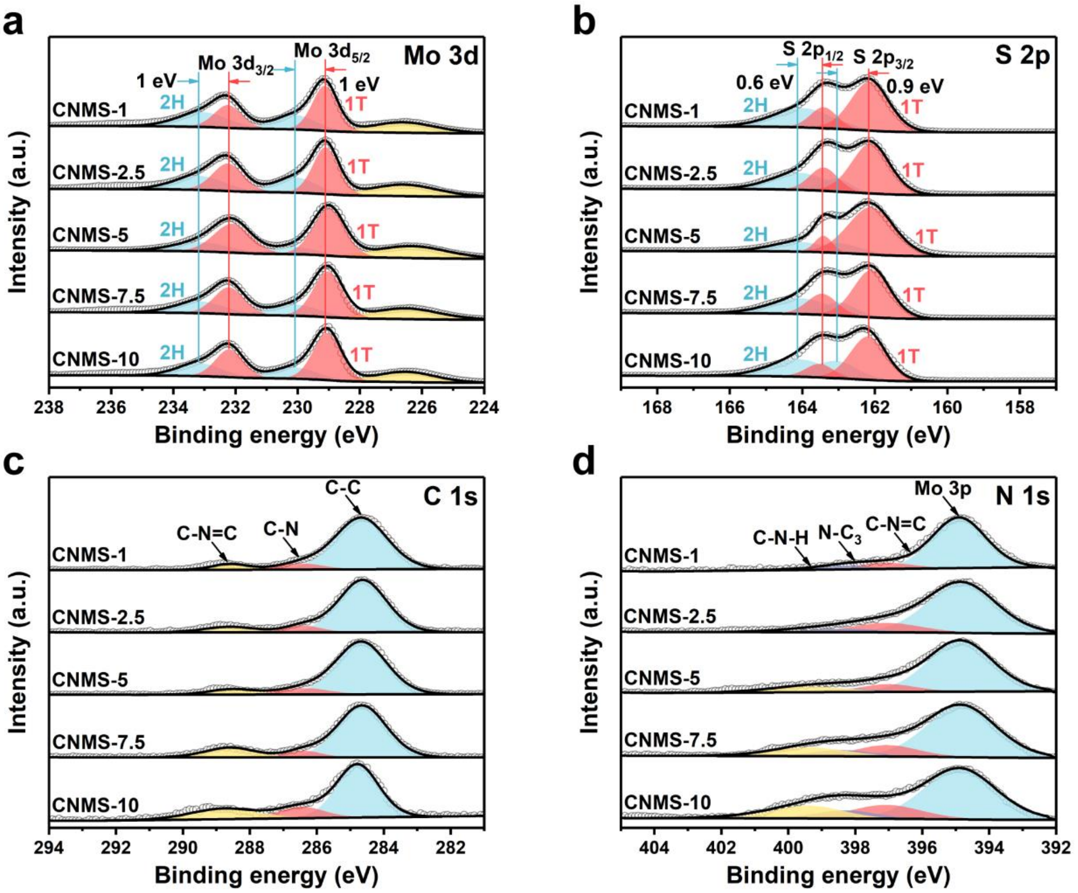

Figure S9. XPS spectra of (a) Mo 3d, (b) S 2p, (c) C 1s, and (d) N 1s of CNMS-1, CNMS-2.5, CNMS-5, CNMS-7.5, and CNMS-10.

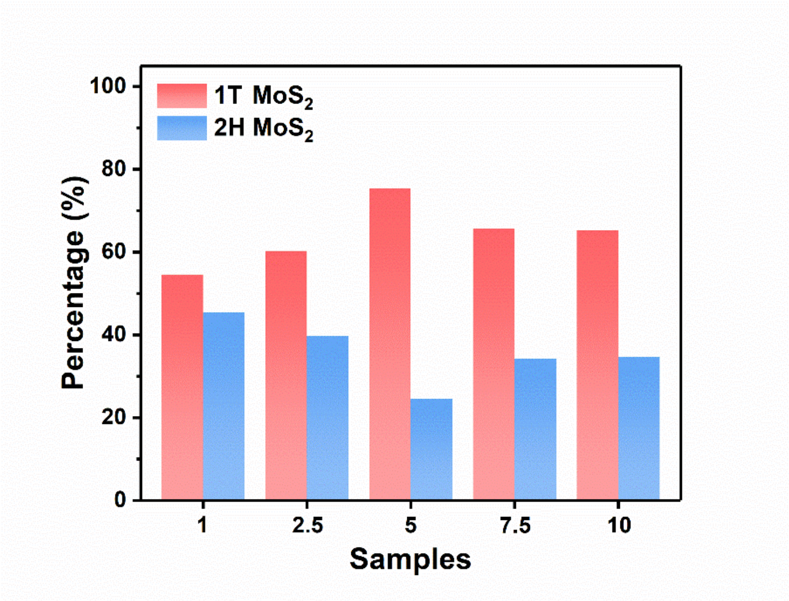

Figure S10. Relative fractions of 1T and 2H phases of CNMS-1, CNMS-2.5, CNMS-5, CNMS-7.5, and CNMS-10.

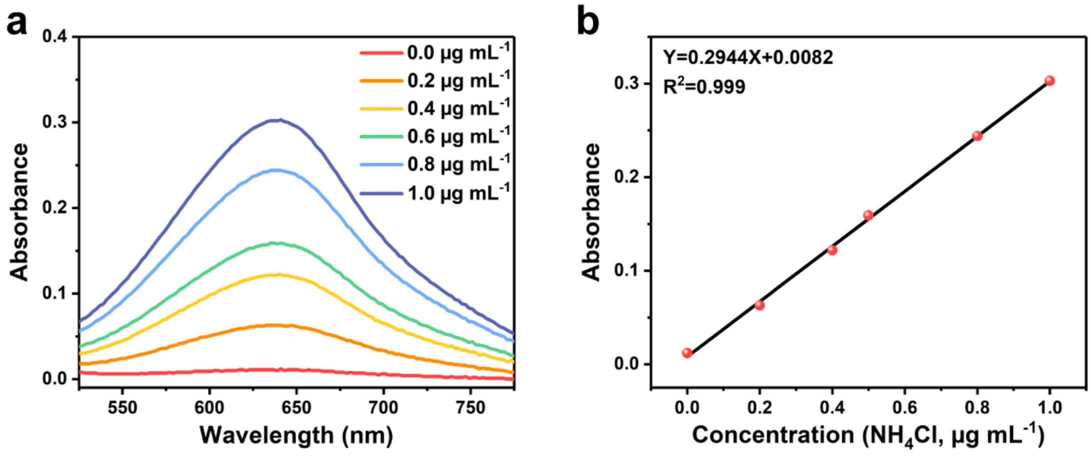

Figure S11. (a) UV-vis spectra of indophenol assays with NH_4^+ ions after being incubated for 30 min at room temperature. (b) Calibration curve for the estimation of NH_4Cl.

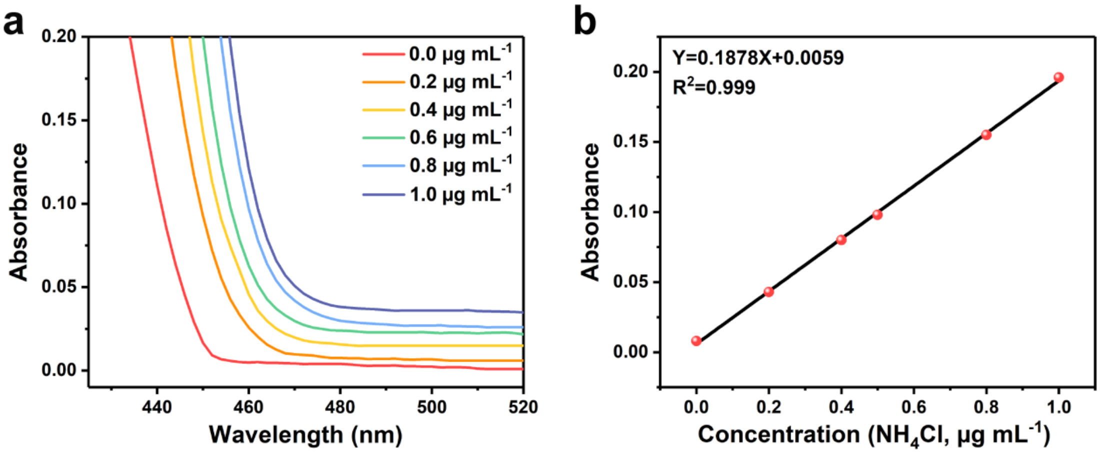

Figure S12. (a) UV-vis spectra of various N_2H_4 concentrations after being incubated for 20 min at room temperature. (b) Calibration curve for the concentration calculation of N_2H_4.

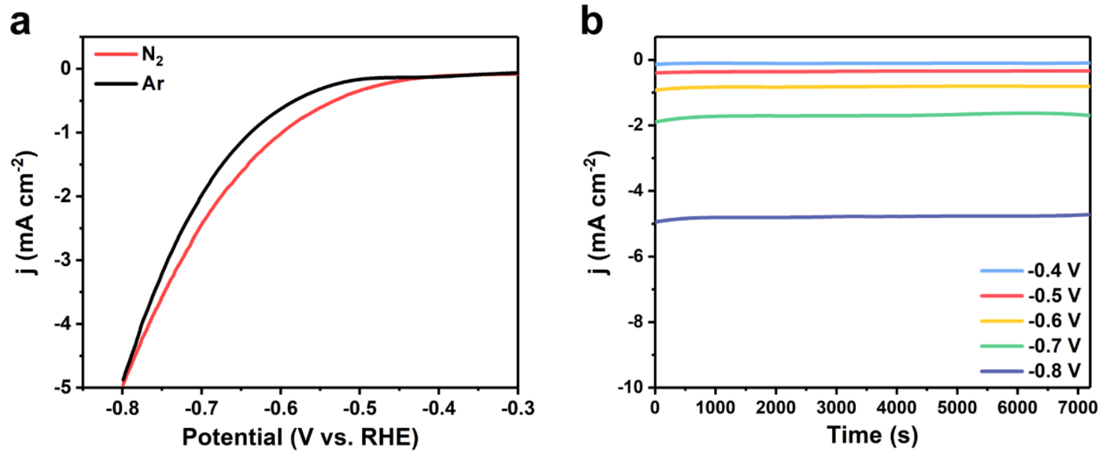

Figure S13. (a) LSV curves of CNMS at N_2- and Ar-saturated 0.1 м Na_2SO_4 electrolytes with a scan rate of 2 mV s^{-1}. (b) Time-current density curves at various potentials.

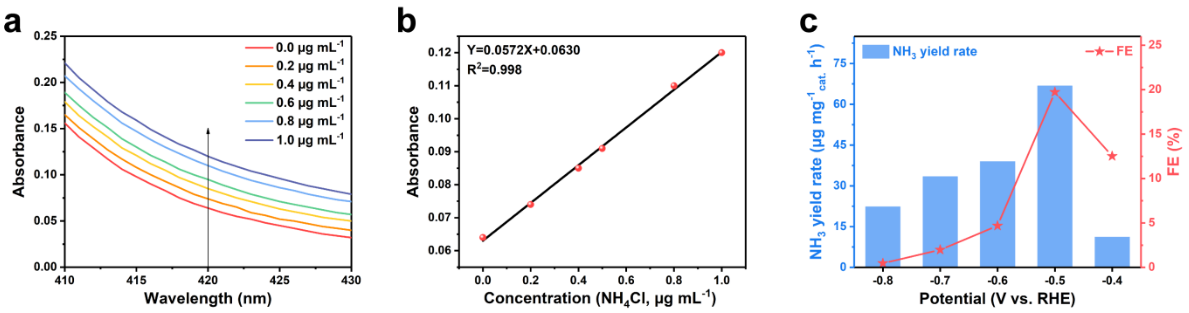

Figure S14. (a) UV-vis spectra of Nessler's reagent assays with NH_4^+ ions after being incubated for 30 min at room temperature. (b) Calibration curve for the estimation of NH_4Cl. (c) NH_3 yield rates and FEs.

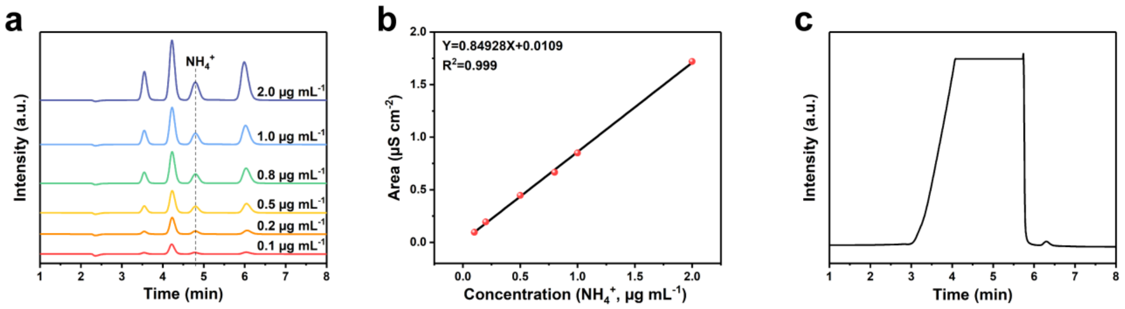

Figure S15. (a) The spectra of ion chromatography for standard solution with various concentrations. (b) Standard curve for NH_3 detection with the ion chromatography method. (c) The spectrum of ion chromatography for the reaction solution over CNMS at -0.5 V for 2 h.

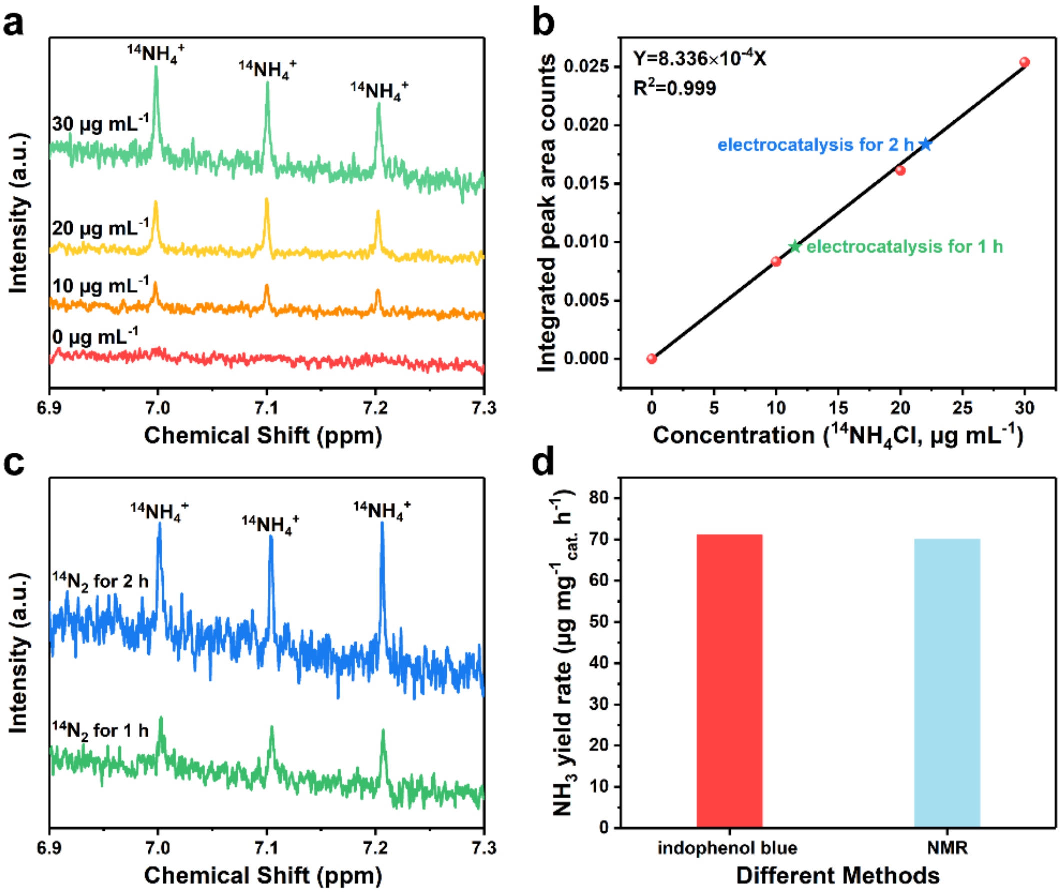

Fig. 16. ^{1}H NMR testing the concentrations of ammonia in the electrochemical process by feeding $^{14}N_2$. (a) ^{1}H NMR spectra for standard $^{14}NH_4Cl$ solutions. (b) Calibration curve of standard $^{14}NH_4Cl$ solutions. (c) ^{1}H NMR spectra for the electrolyte fed by $^{14}N_2$ over CNMS after reaction for 1 h and 2 h at -0.5 V vs. RHE. (d) Comparison of the $^{14}NH_4^+$ yield rate in the electrochemical process detected by the indophenol blue method and NMR.

Table S2. The NRR activity comparison over various aqueous electrocatalysts at ambient conditions.

**Catalyst**	**Electrolyte**	**NH_3 yield**	**FE (%)**	**Ref.**
CNMS	0.1 м Na_2SO_4	71.07 μg h^{-1} $mg_{cat.}^{-1}$	21.01	This work
1 T-MoS_2/g-C_3N_4	0.1 м HCl	29.97 μg h^{-1} $mg_{cat.}^{-1}$	20.48	[20]
1T-MoS_2@Ti_3C_2	0.1 м HCl	30.33 μg h^{-1} $mg_{cat.}^{-1}$	10.94	[21]
1T-MoS_2 /BTAB/PPy/GO	0.1 м KOH	7.05 μg h^{-1} $mg_{cat.}^{-1}$	5.88	[22]
Ru/MoS_2	0.1 м HCl	1.14×10^{-10} mo1 s^{-1} cm^{-2}	17.60	[23]
Defect-rich MoS_2 nanoflower	0.1 м Na_2SO_4	29.28 μg h^{-1} $mg_{cat.}^{-1}$	8.34	[24]
MoS_2-rGO	0.1 м $LiClO_4$	24.82 μg h^{-1} $mg_{cat.}^{-1}$	4.58	[25]
MoS_2 nanosheet	0.1 м Na_2SO_4	13.09 μg h^{-1} $mg_{cat.}^{-1}$	1.17	[26]
MoS_2/C_3N_4	0.1 м $LiClO_4$	18.50 μg h^{-1} $mg_{cat.}^{-1}$	17.80	[27]
Co-doped MoS_{2-x}	0.01 м H_2SO_4	0.63 mmol g^{-1} h^{-1}	10	[28]
MoS_2-PDR	0.1 м K_2SO_4	43.40 μg h^{-1} $mg_{cat.}^{-1}$	16.80	[29]
Sulfur vacancy-rich N-doped MoS_2 nanoflower	0.1 м Na_2SO_4	69.82 μg h^{-1} $mg_{cat.}^{-1}$	9.14	[30]
Fe-MoS_2 nanosheets	0.5 м K_2SO_4	8.63 μg h^{-1} $mg_{cat.}^{-1}$	18.80	[31]
(110)-oriented Mo	0.5 м H_2SO_4	3.09×10^{-11} mo1 s^{-1} cm^{-2}	0.72	[32]

nanofilm				
MoSAs-Mo_2C/NCNTs	0.5 м H_2SO_4	16.10 μg h^{-1} $mg_{cat.}^{-1}$	7.10	[33]
Mo_2C nanorod	0.1 м HCl	95.10 μg h^{-1} $mg_{cat.}^{-1}$	8.13	[34]
Mo_2C/C	0.5 м Li_2SO_4	11.30 μg h^{-1} $mg_{cat.}^{-1}$	7.80	[35]
MoN nanosheet	0.1 м HCl	3.01×10^{-10} mo1 s^{-1} cm^{-2}	1.15	[36]
Mo_2N nanorod	0.1 м HCl	78.40 μg h^{-1} $mg_{cat.}^{-1}$	4.50	[37]
MoO_3 nanosheets	0.1 м HCl	29.43 μg h^{-1} $mg_{cat.}^{-1}$	1.90	[38]
WO_3 nanosheets	0.1 м HCl	17.28 μg h^{-1} $mg_{cat.}^{-1}$	7.00	[39]
VO_2 hollow microsphere	0.1 м Na_2SO_4	14.85 μg h^{-1} $mg_{cat.}^{-1}$	3.97	[40]
PCN	0.1 м HCl	8.09 μg h^{-1} $mg_{cat.}^{-1}$	11.59	[41]
V_2O_3/C	0.1 м Na_2SO_4	12.30 μg h^{-1} $mg_{cat.}^{-1}$	7.28	[42]
TiO_2-rGO	0.1 м Na_2SO_4	15.13 μg h^{-1} $mg_{cat.}^{-1}$	3.30	[43]
Fe-N/C-CNTs	0.1 м KOH	34.83 μg h^{-1} $mg_{cat.}^{-1}$	9.28	[44]
$Ti_3C_2T_x$ nanosheet	0.1 м HCl	20.40 μg h^{-1} $mg_{cat.}^{-1}$	9.30	[45]
Pd-Co/CuO	0.1 м KOH	10.04 μg h^{-1} $mg_{cat.}^{-1}$	2.16	[46]
Hollow Cr_2O_3 microspheres	0.1 м Na_2SO_4	25.30 μg h^{-1} $mg_{cat.}^{-1}$	6.78	[47]
FeN_4	0.1 м Na_2SO_4	29.43 μg h^{-1} $mg_{cat.}^{-1}$	1.90	[48]
Oxygen-doped carbon nanosheet	0.1 м HCl	20.15 μg h^{-1} $mg_{cat.}^{-1}$	4.97	[49]
Pd/C	0.1 м PBS	4.50 μg h^{-1} $mg_{cat.}^{-1}$	8.20	[50]

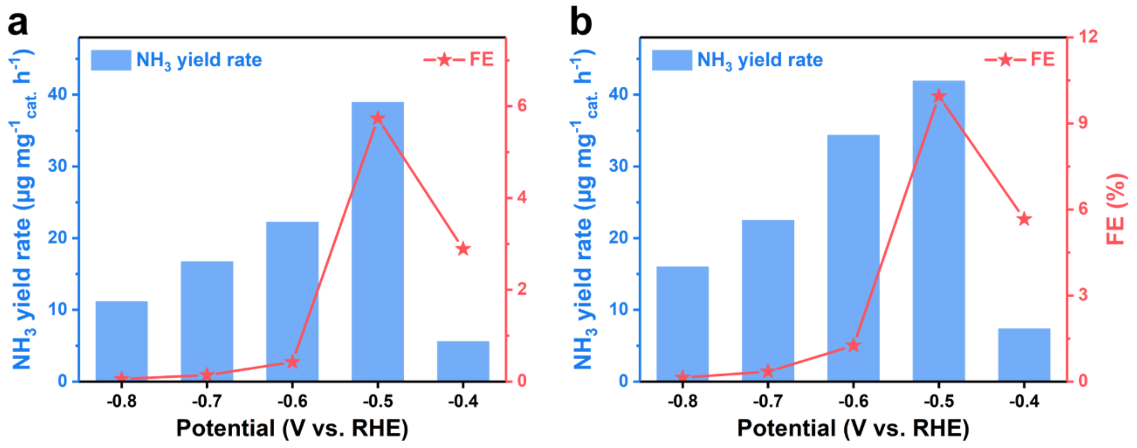

Figure S17. NH_3 yield rates and FEs of CNMS after electrocatalysis in (a) 0.1 м HCl and (b) 0.1 м KOH.

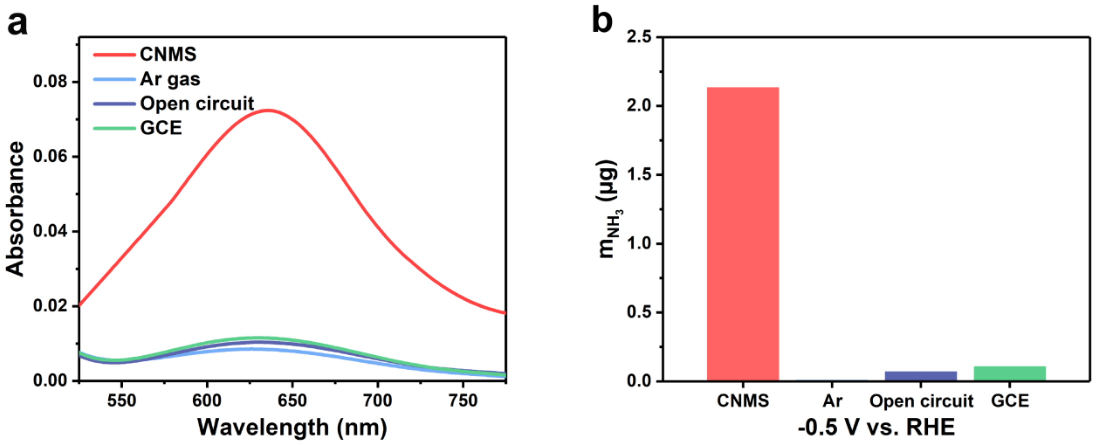

Figure S18. (a) UV-vis absorption spectra of the electrolytes stained with indophenol indicator. (b) Amount of NH_3 generated after charging at -0.5 V for 2 h under various conditions.

Four extra control experiments were performed: (1) CNMS in a N_2-saturated 0.1 м Na_2SO_4 solution at an open circuit potential for 2 h; (2) CNMS in an Ar-saturated 0.1 м Na_2SO_4 solution at -0.5 V for 2 h; (3) a bare GCE in a N_2-saturated 0.1 м Na_2SO_4 solution at -0.5 V for 2 h; (4) CNMS at -0.5 V with alternating 2 h cycles between N_2- and Ar- saturated 0.1 м Na_2SO_4 solution for a total of 12 h.

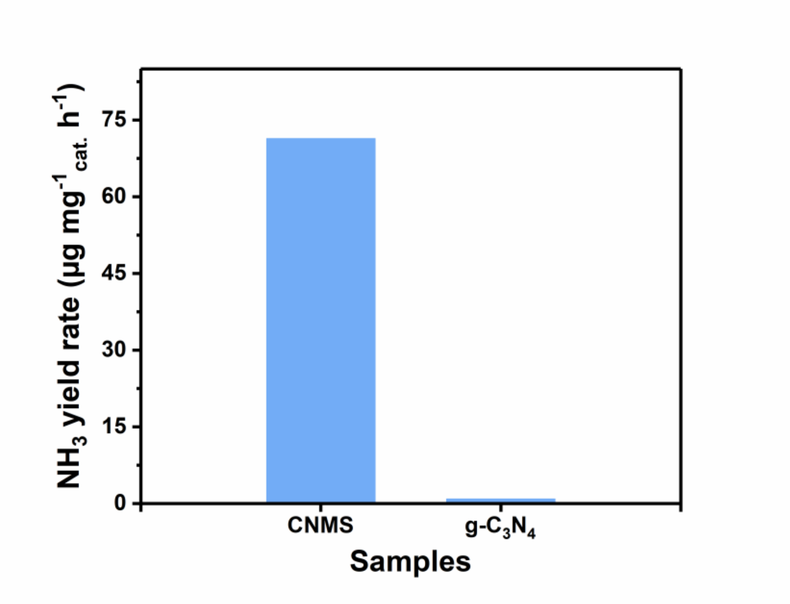

Figure S19. NH_3 yield rates at -0.5 V of CNMS and g-C_3N_4.

To eliminate the effect of g-C_3N_4 that may not be fully dissolved during the hydrothermal process on NRR activities, the performance of g-C_3N_4 was also evaluated. It is worth noting that bare g-C_3N_4 displays negligible NRR activities (NH_3 yield rate: 0.86 μg h^{-1} $mg^{-1}_{cat.}$), revealing that the g-C_3N_4 has almost no catalytic activity for the NRR.

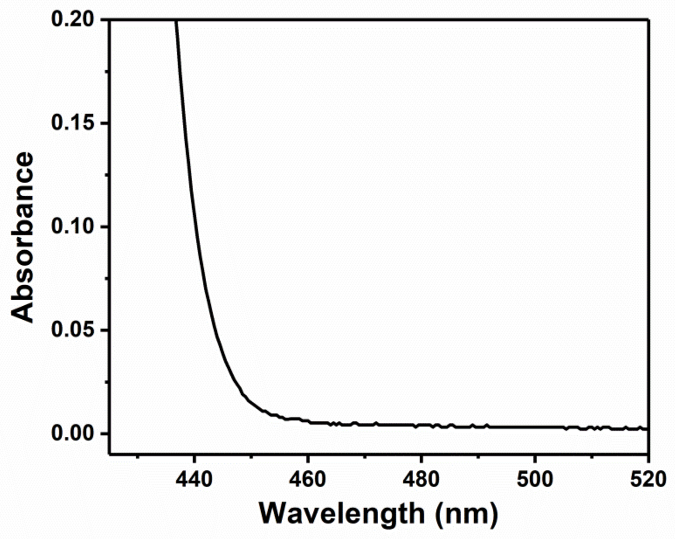

Figure S20. UV-vis absorption spectrum of CNMS charging at -0.5 V vs. RHE after being incubated with the N_2H_4 color agent for 20 min at room temperature.

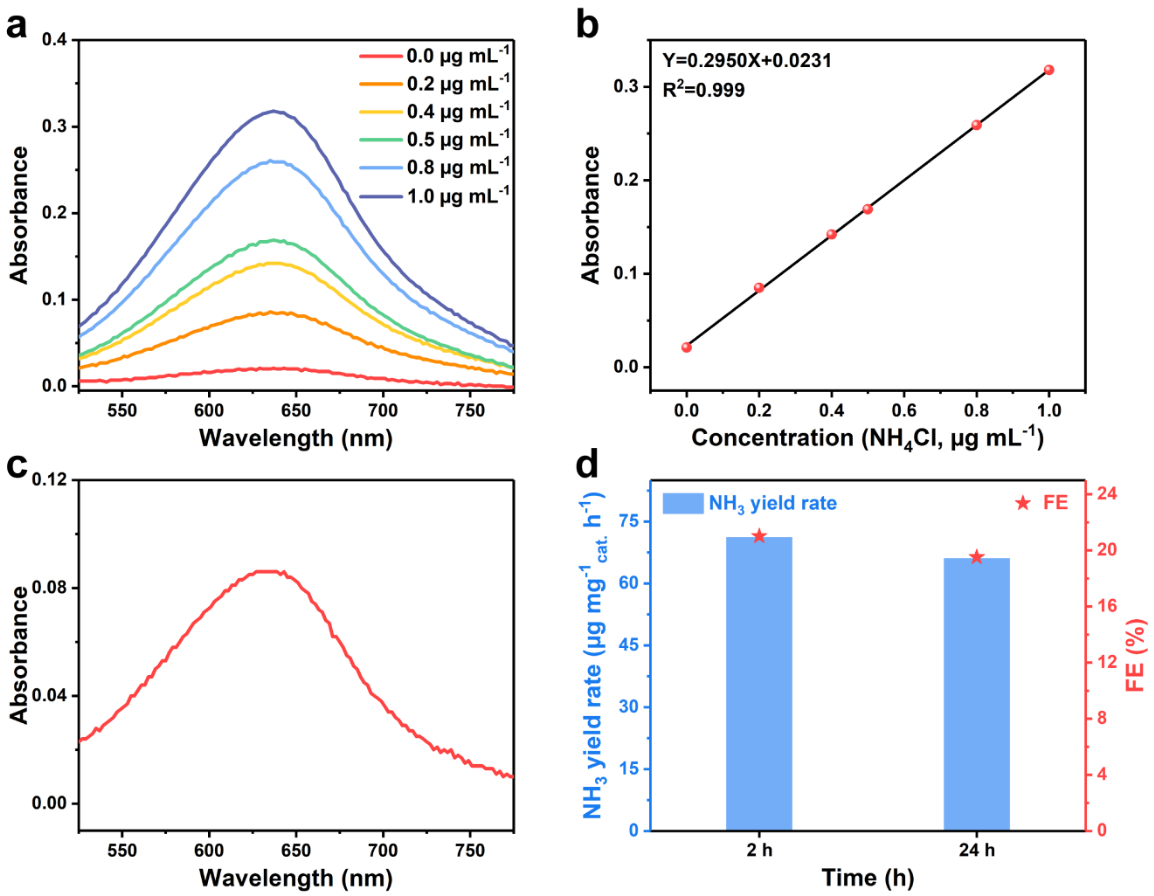

Figure S21. (a) UV-vis spectra of indophenol assays with NH_4^+ ions after being incubated for 30 min at room temperature. (b) Calibration curve for the estimation of NH_4Cl. (c) UV-vis spectra of indophenol assays with NH_4^+ ions after electrocatalysis for 24 h. (d) The NRR performance of CNMS after electrocatalysis for 2 h and 24 h.

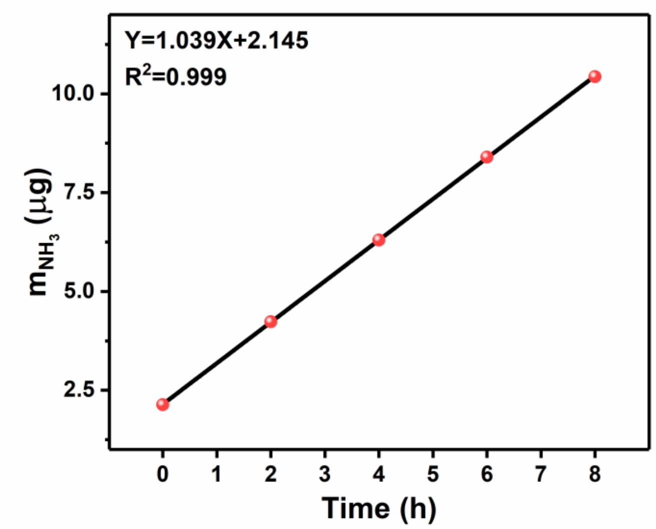

Figure S22. Curve of ammonia yield *vs* reaction time at -0.5 V.

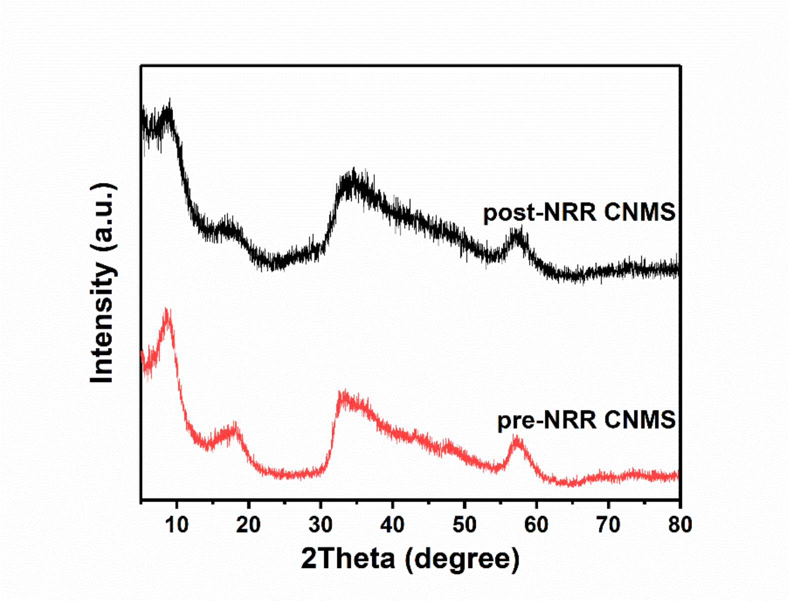

Figure S23. XRD patterns of CNMS before and after the NRR electrolysis.

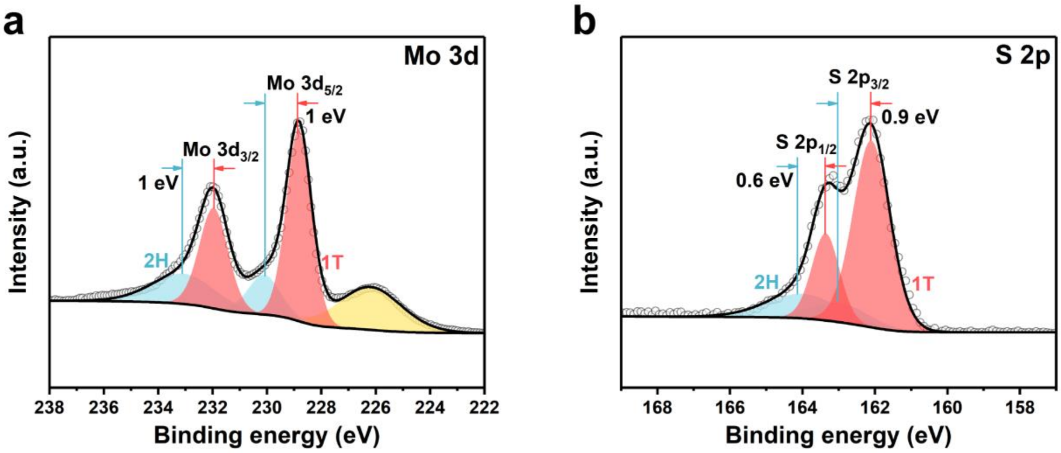

Figure S24. XPS spectra of (a) Mo 3d and (b) S 2p over CNMS after the NRR.

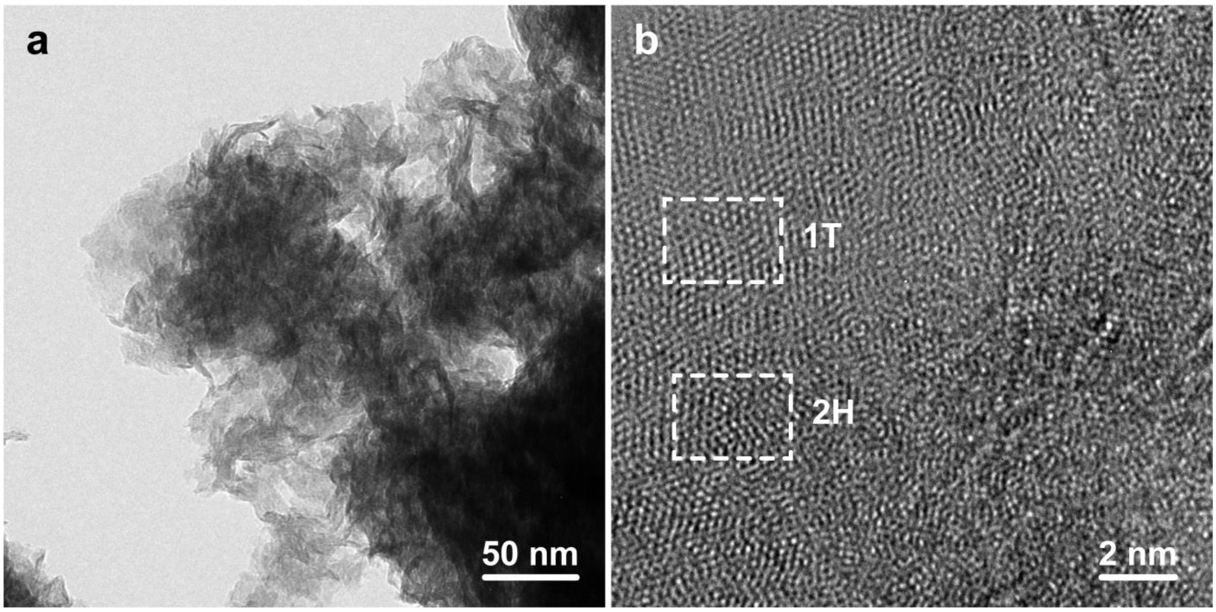

Figure S25. (a) TEM and (b) HRTEM images of CNMS after the NRR.

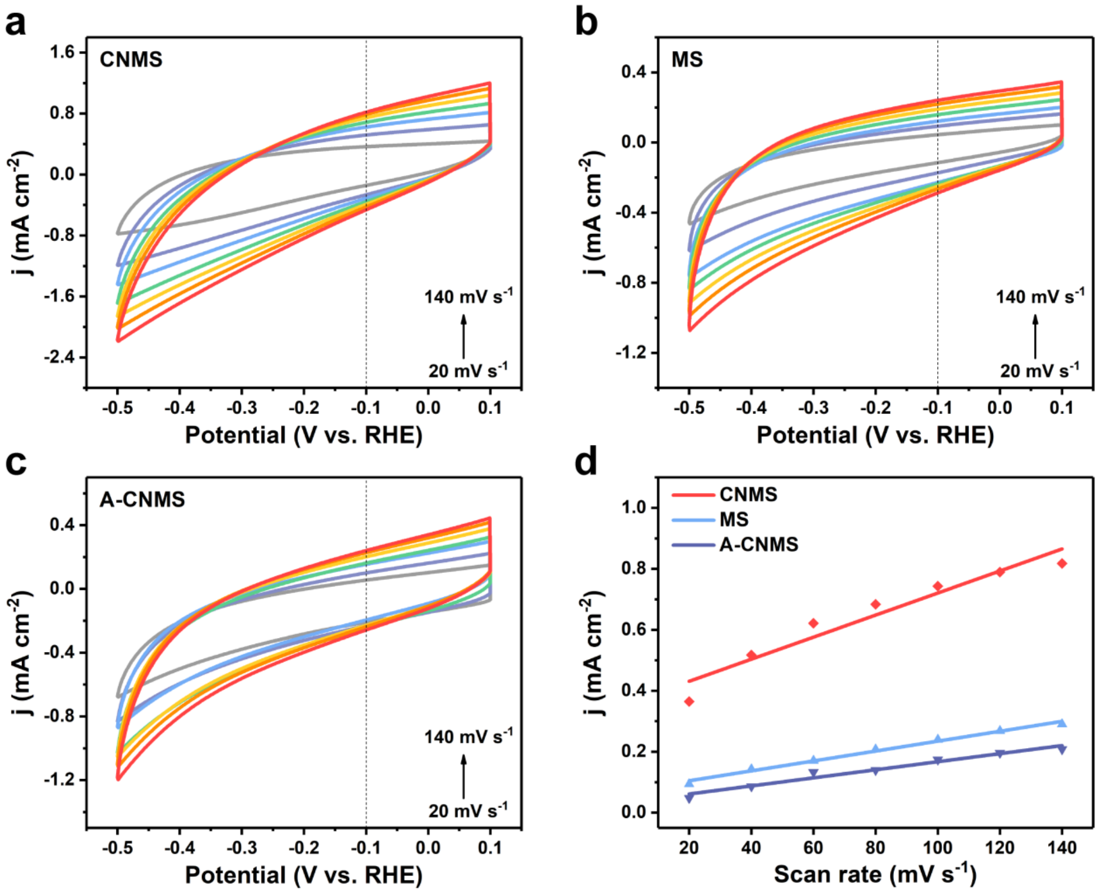

Figure S26. CVs of (a) CNMS, (b) MS, and (c) A-CNMS. (d) Measured capacitive currents plotted as a function of scan rate.

Table S3. The C_{dl} and electrochemically active surface area of various catalysts.

Catalysts	CNMS	MS	A-CNMS
C_{dl} (μF cm^{-2})	1810	810	660
ECSA (cm^2)	30.2	13.5	11.0

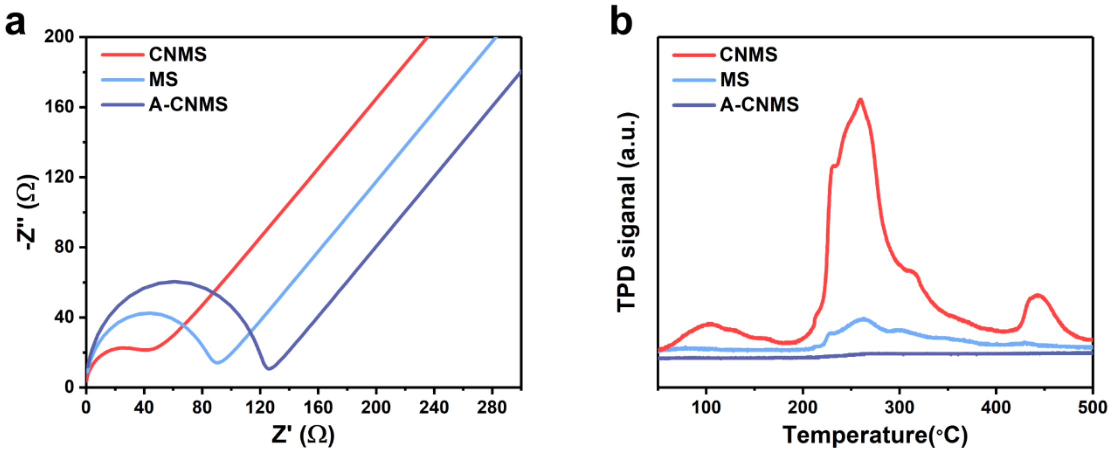

Figure S27. (a) Nyquist plots and (b) Nitrogen temperature-programmed desorption of CNMS, MS, and A-CNMS.

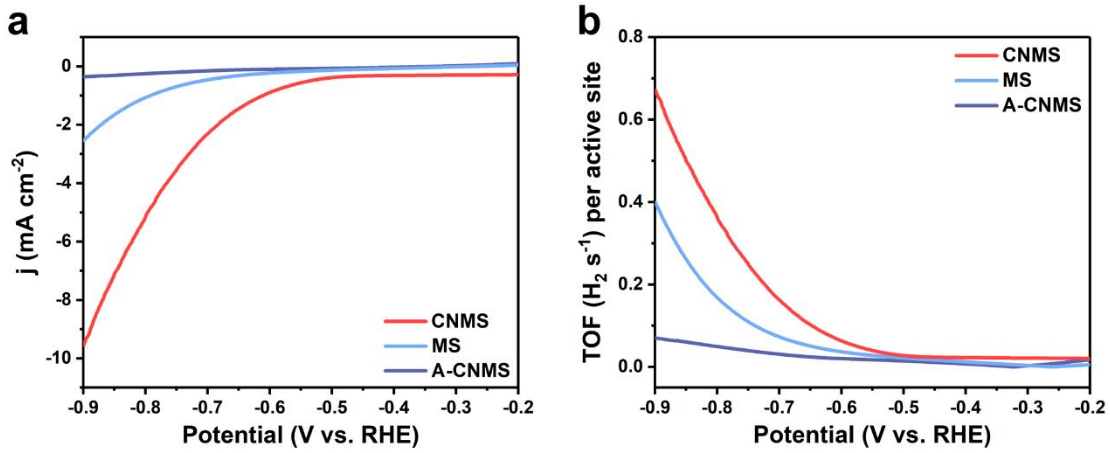

Figure S28. (a) LSV curves of all samples in Ar-saturated 0.1 м Na_2SO_4 solution. (b) Evolution of the TOF_{HER} with the applied potential.

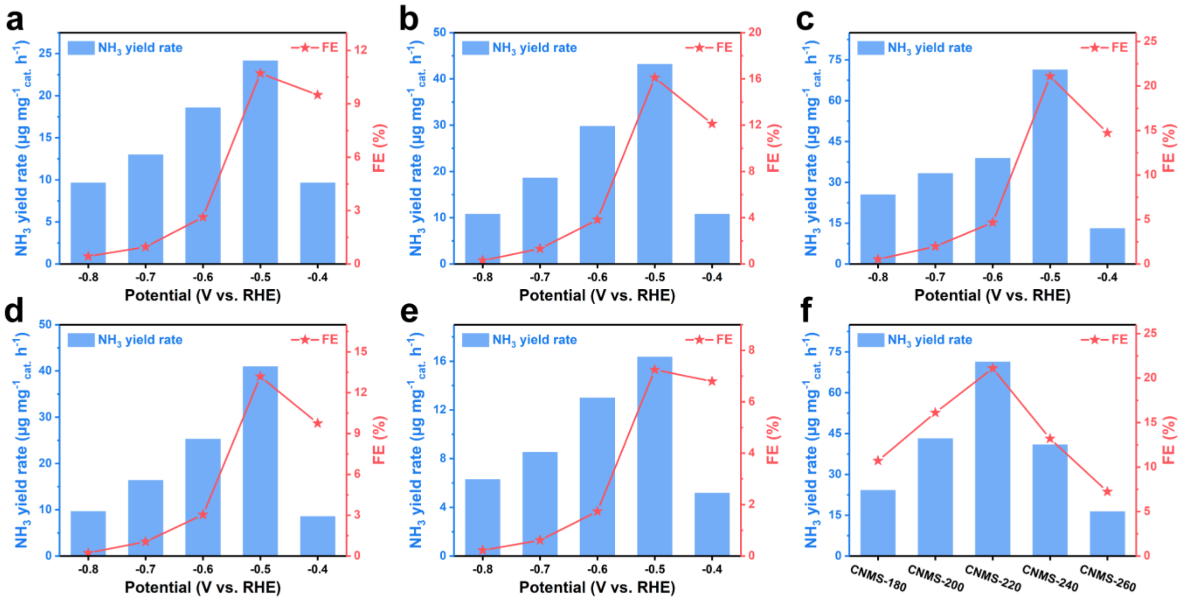

Figure S29. NH_3 yield rates and corresponding FEs of (a) CNMS-180, (b) CNMS-200, (c) CNMS-220, (d) CNMS-240, and (e) CNMS-260 at -0.5 V vs. RHE for above catalysts under the same conditions. (f) Comparison of the properties over catalysts at various temperatures.

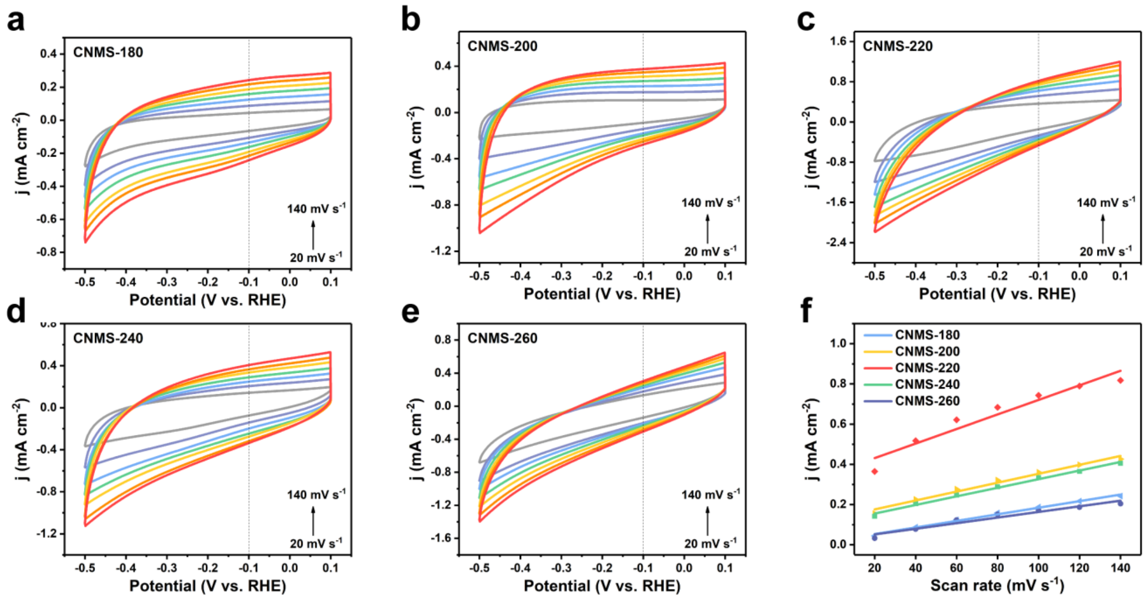

Figure S30. CVs of (a) CNMS-180, (b) CNMS-200, (c) CNMS-220, (d) CNMS-240, and (e) CNMS-260. (f) Measured capacitive currents plotted as a function of scan rate.

Table S4. The C_{dl} and electrochemically active surface area of various catalysts.

Catalysts	CNMS-180	CNMS-200	CNMS-220	CNMS-240	CNMS-260
C_{dl} (μF cm^{-2})	815	1110	1810	1070	700
ECSA (cm^2)	13.6	18.5	30.2	17.8	11.7

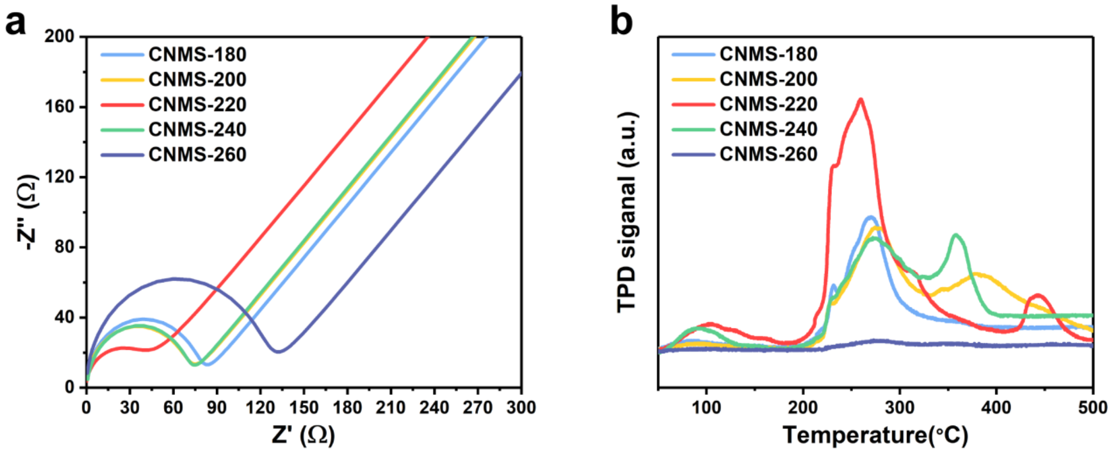

Figure S31. (a) Nyquist plots and (b) N_2 adsorption curves of CNMS-180, CNMS-200, CNMS-220, CNMS-240, and CNMS-260.

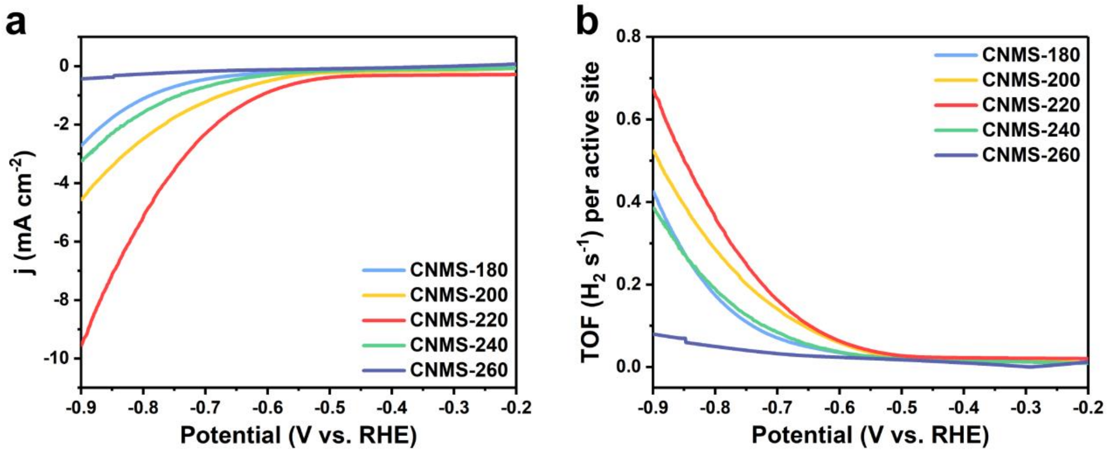

Figure S32. (a) LSV curves of all samples in Ar-saturated 0.1 м Na_2SO_4 solution. (b) Evolution of the TOF_{HER} with the applied potential.

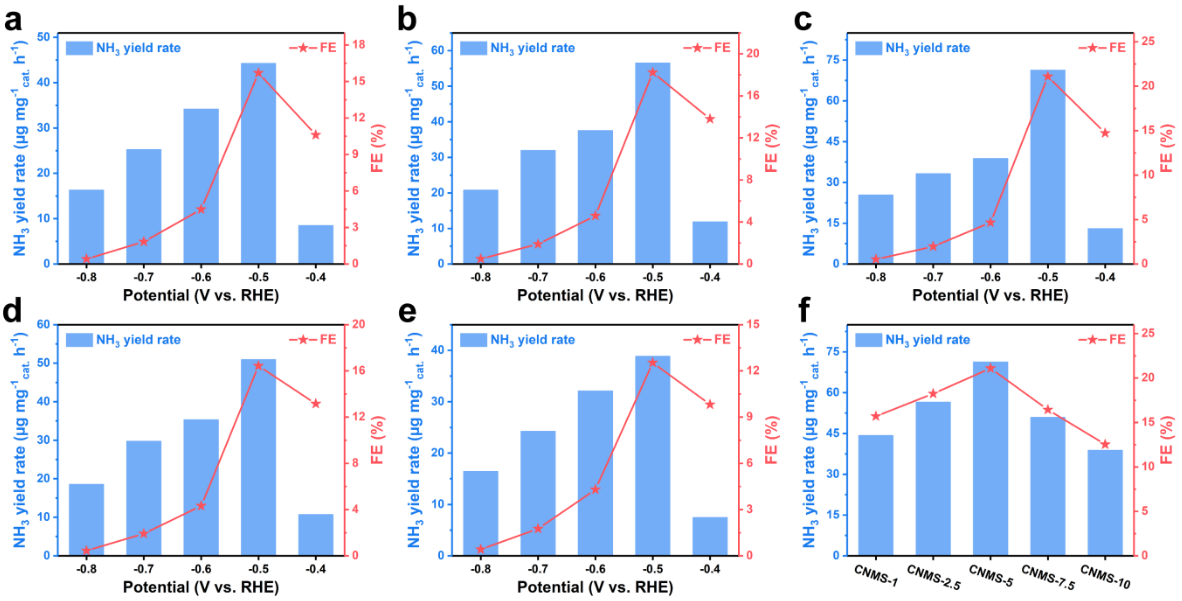

Figure S33. NH_3 yield rates and corresponding FEs of (a) CNMS-1, (b) CNMS-2.5, (c) CNMS-5, (d) CNMS-7.5, and (e) CNMS-10. (f) Comparison of properties over catalysts obtained with various g-C_3N_4/MoS_2 ratios.

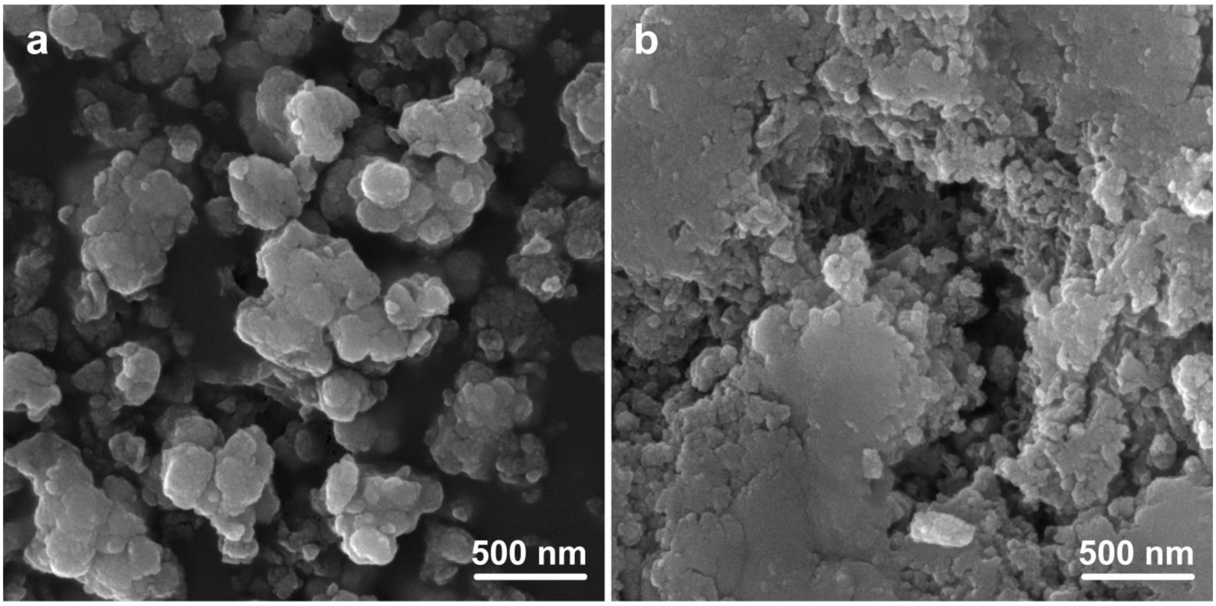

Figure S34. SEM images of (a) CNMS-7.5 and (b) CNMS-10.

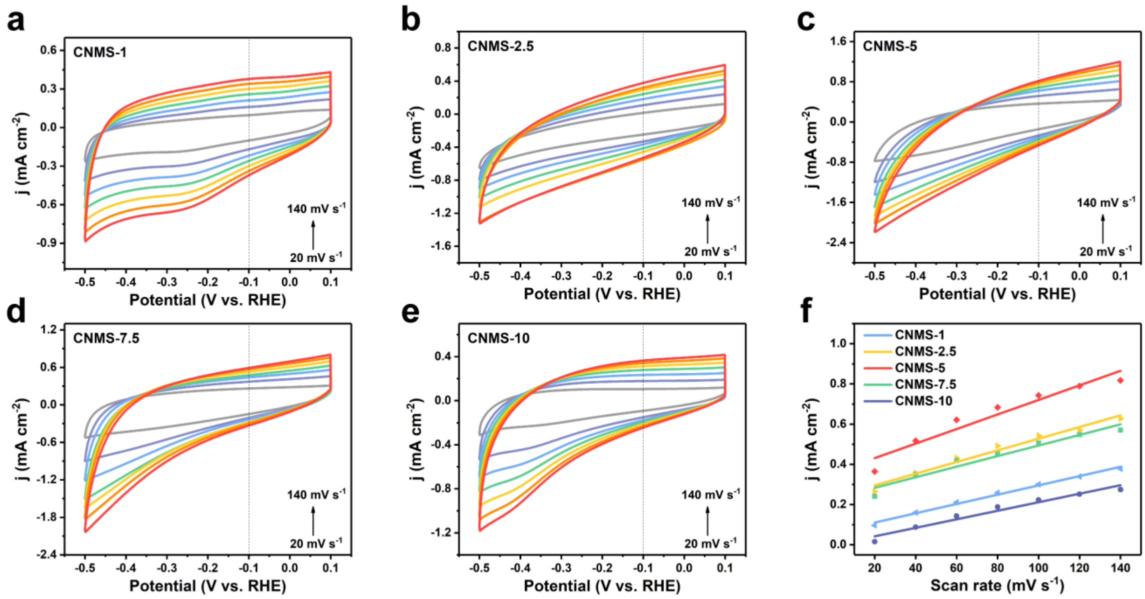

Figure S35. CVs of (a) CNMS-1, (b) CNMS-2.5, (c) CNMS-5, (d) CNMS-7.5, and (e) CNMS-10, and (e) CNMS-260. (f) Measured capacitive currents plotted as a function of scan rate.

Table S5. The C_{dl} and electrochemically active surface area of various catalysts.

Catalysts	CNMS-1	CNMS-2.5	CNMS-5	CNMS-7.5	CNMS-10
C_{dl} (μF cm^{-2})	1155	1453	1810	1315	1061
ECSA (cm^2)	19.3	24.2	30.2	21.9	17.7

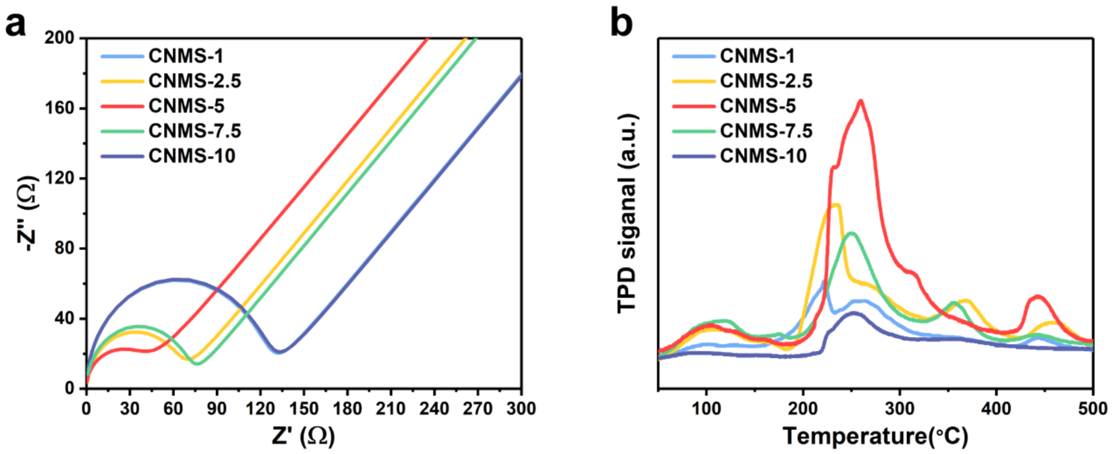

Figure S36. (a) Nyquist plots and (b) N_2 adsorption curves of CNMS-1, CNMS-2.5, CNMS-5, CNMS-7.5, and CNMS-10.

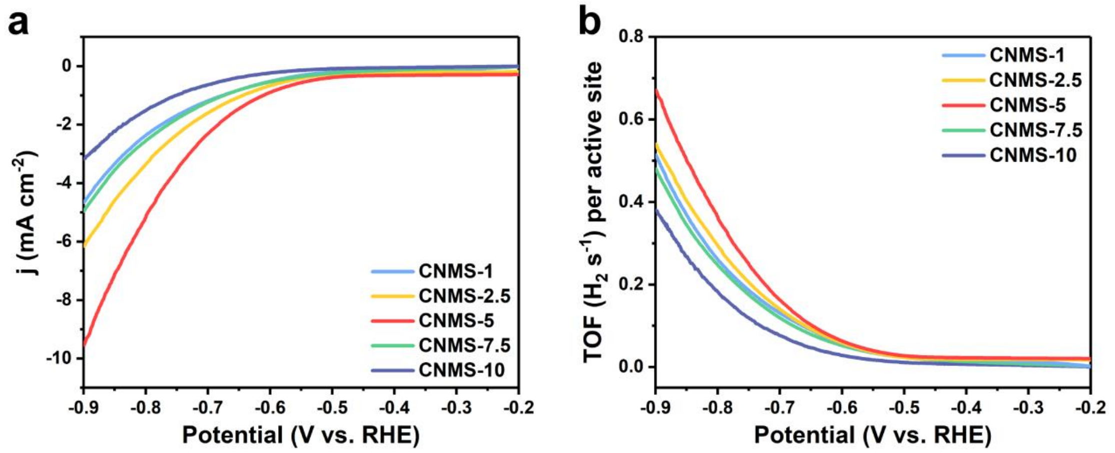

Figure S37. (a) LSV curves of all samples in Ar-saturated 0.1 M Na_2SO_4 solution. (b) Evolution of the TOF_{HER} with the applied potential.

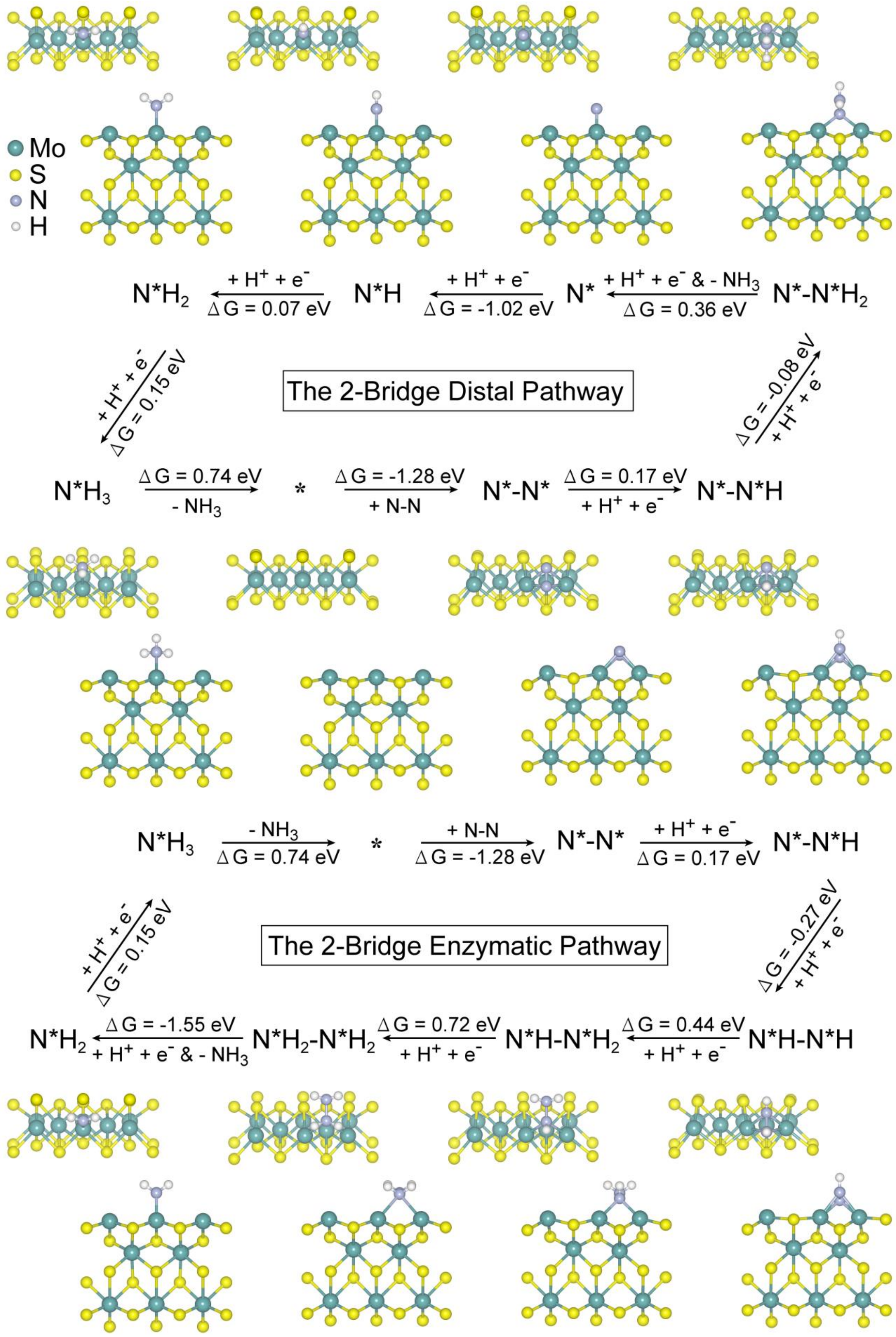

Figure S38. The top and front view of the 1T-MoS_2 with and without NRR intermediates adsorbed along the 2-bridge distal and enzymatic pathway. The Gibbs free energy changes are marked.

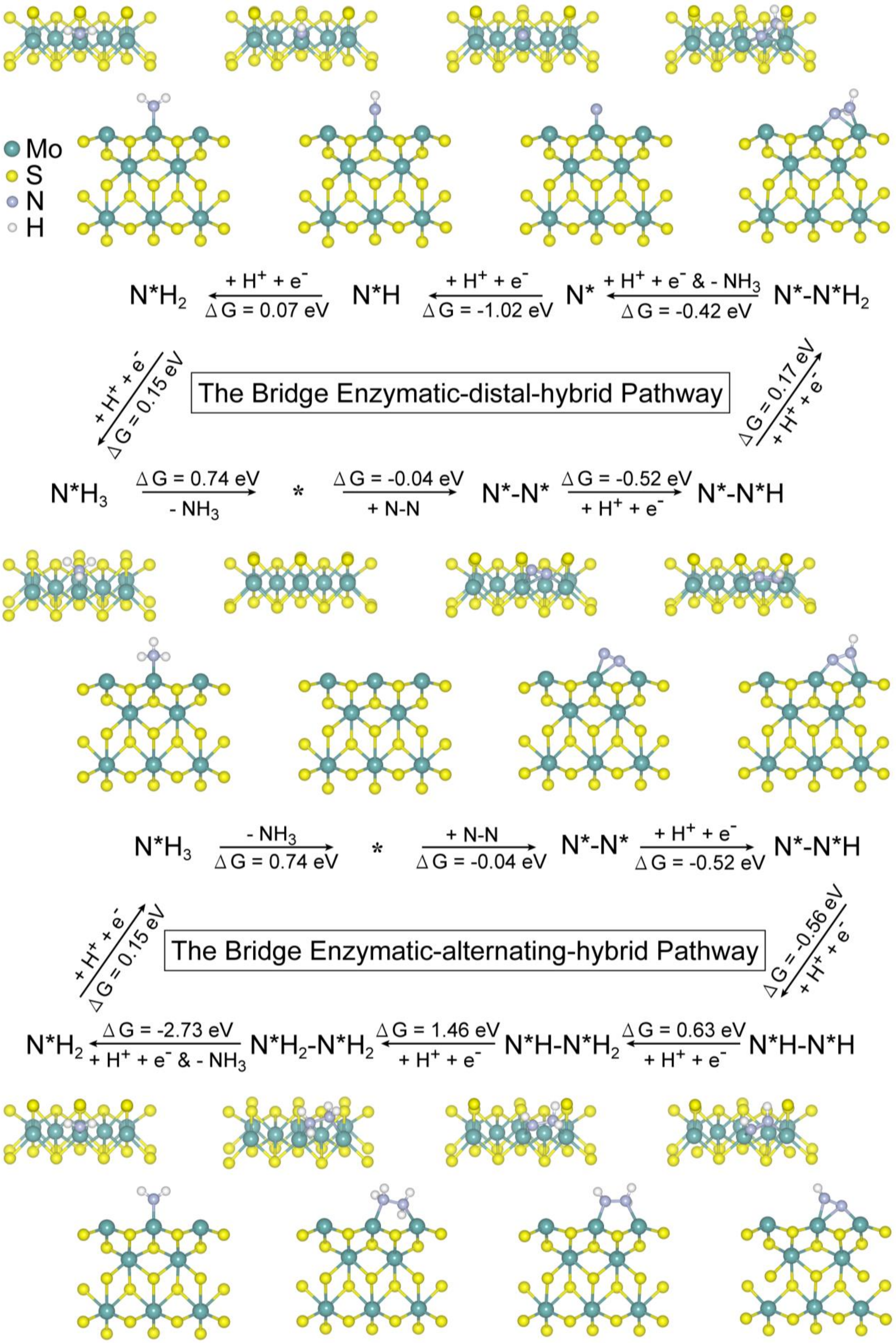

Figure S39. The top and front view of the 1T-MoS_2 with and without NRR intermediates adsorbed along the bridge enzymatic-distal-hybrid and enzymatic-alternating-hybrid pathway. The Gibbs free energy changes are marked.

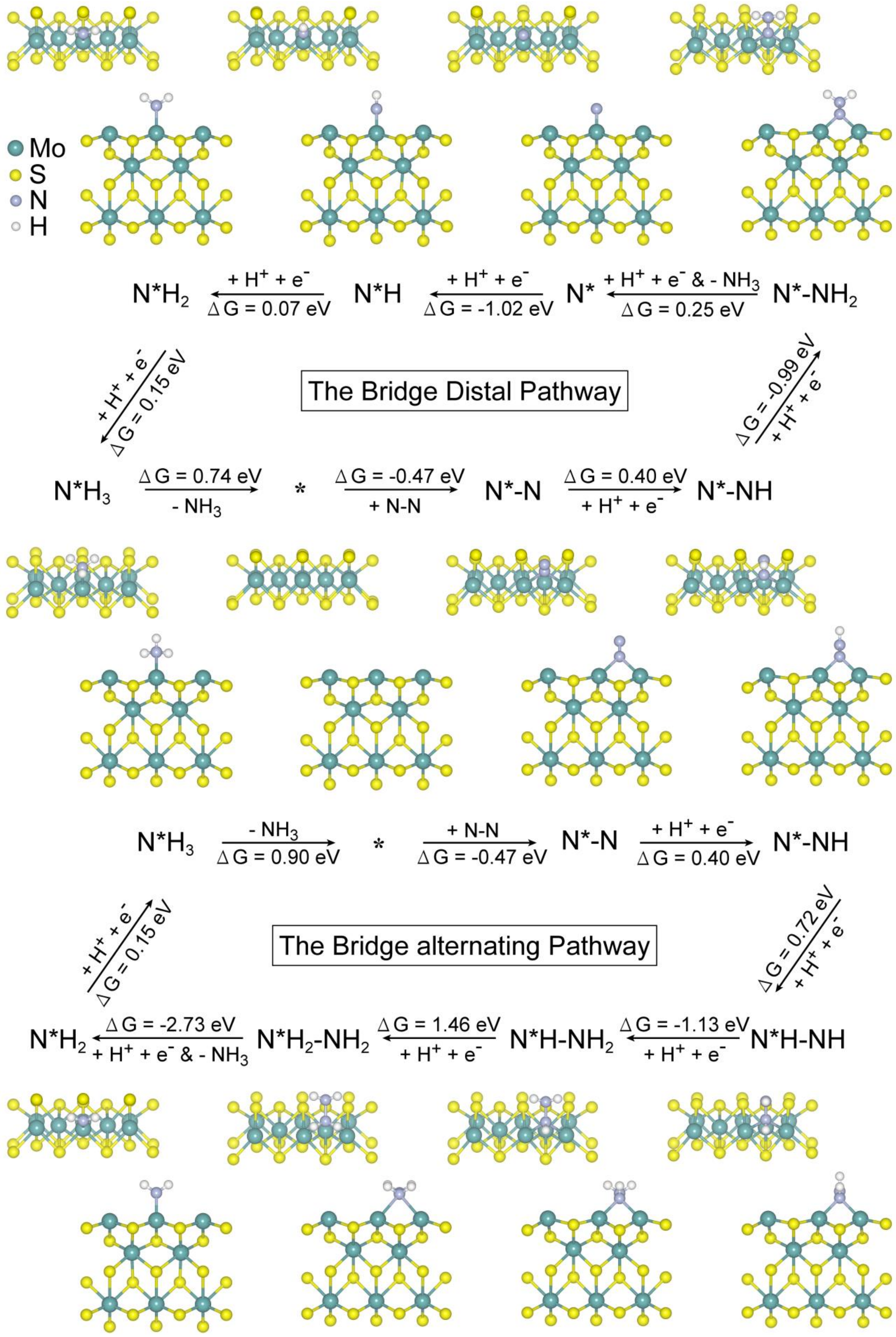

Figure S40. The top and front view of the 1T-MoS_2 with and without NRR intermediates adsorbed along the bridge distal and alternating pathway. The Gibbs free energy changes are marked.

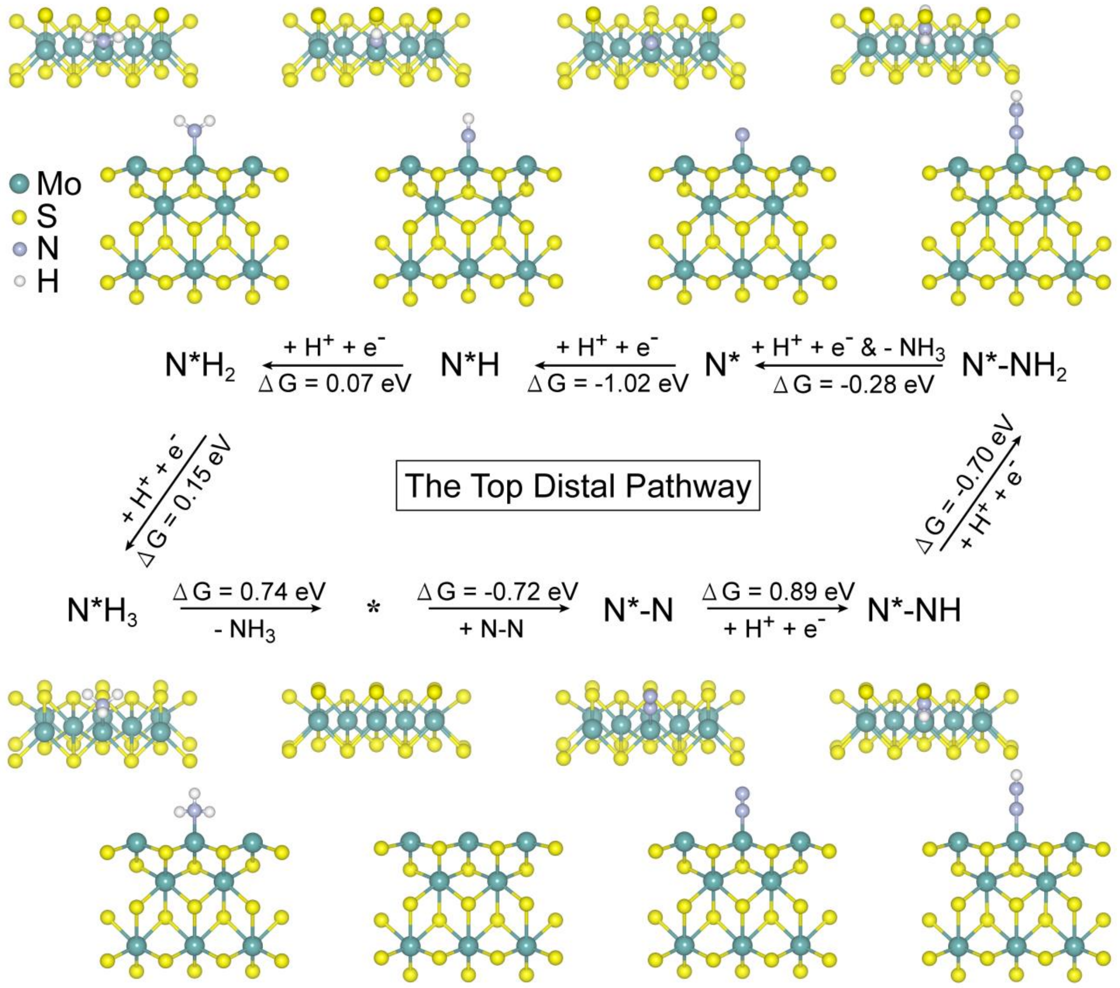

Figure S41. The top and front view of the 1T-MoS_2 with and without NRR intermediates adsorbed along the top distal pathway. The Gibbs free energy changes are marked.

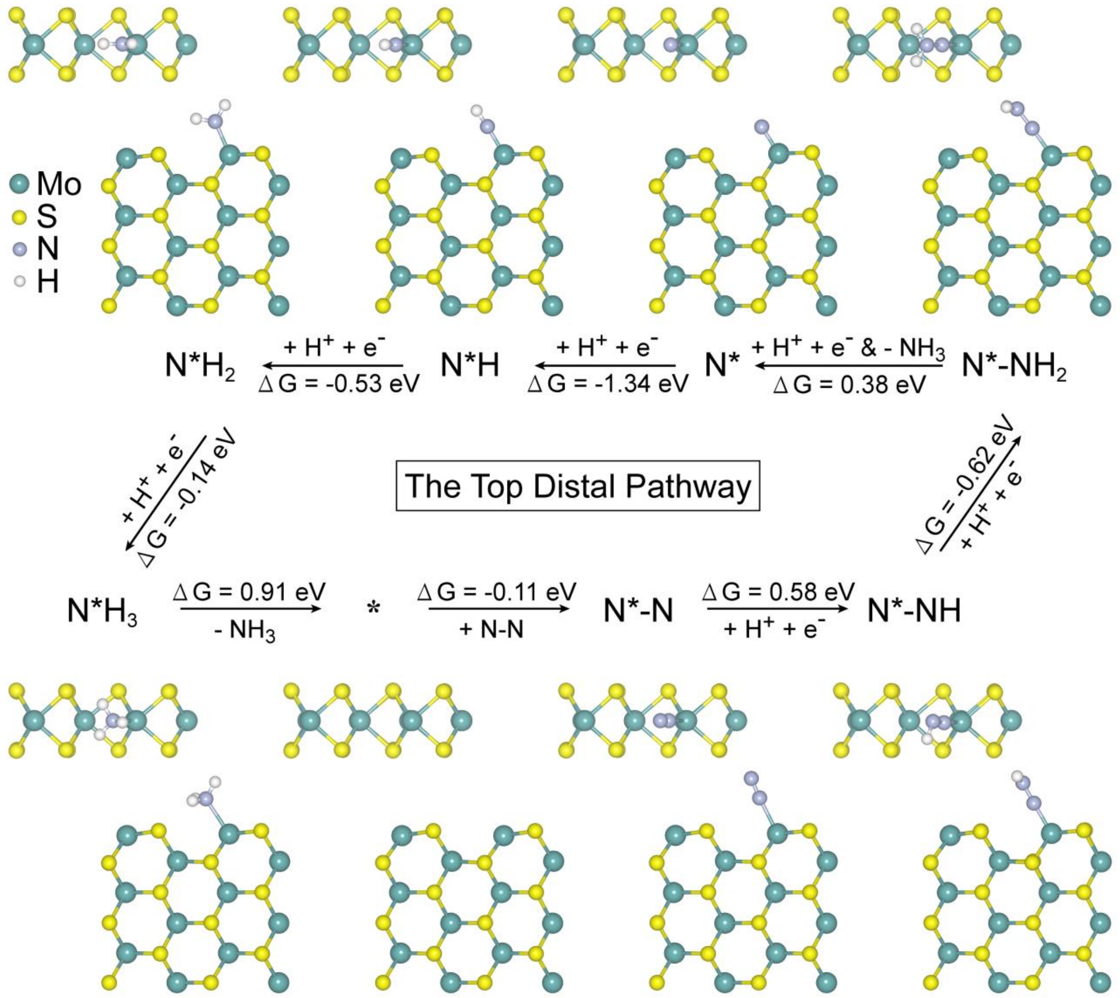

Figure S42. The top and front view of the 2H-MoS_2 with and without NRR intermediates adsorbed along the top distal pathway. The Gibbs free energy changes are marked.

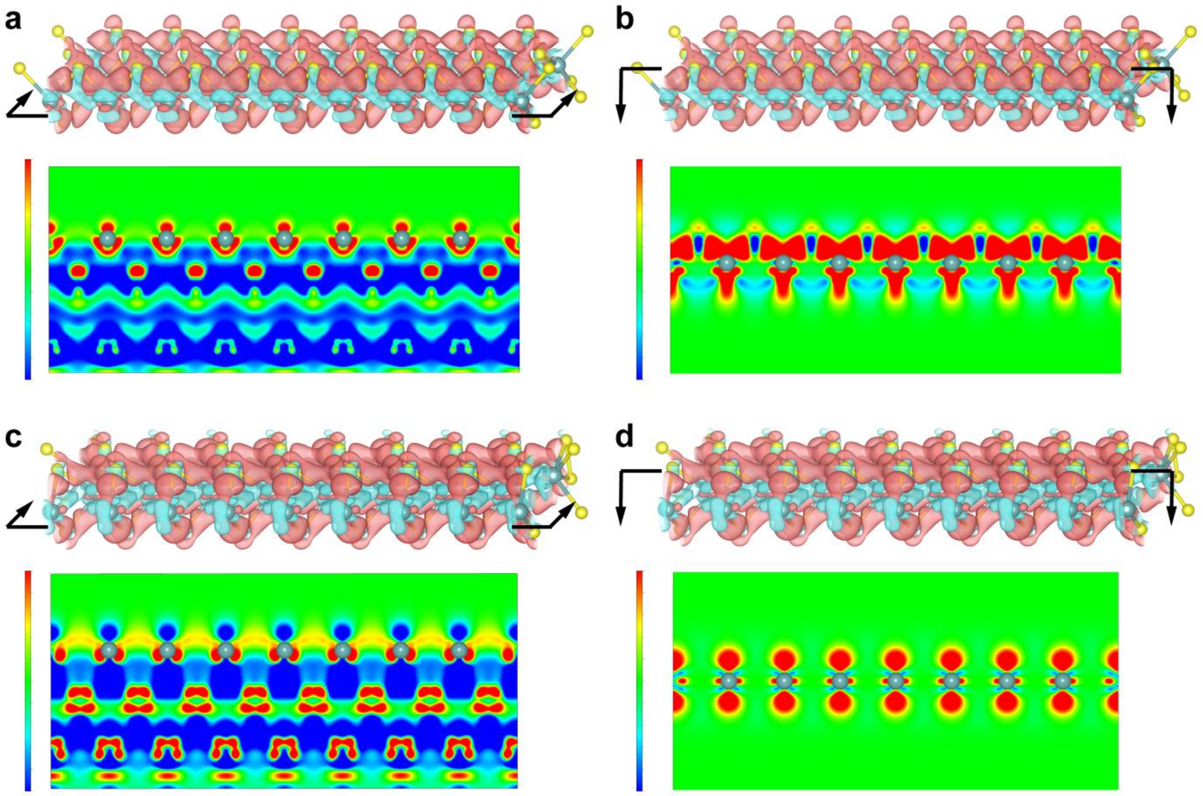

Figure S43. Charge density difference of (a, b) 1T-MoS_2 and (c, d) 2H-MoS_2 and their corresponding sectional drawing. Electron excess and deficiency were represented as red and blue isosurfaces, respectively.

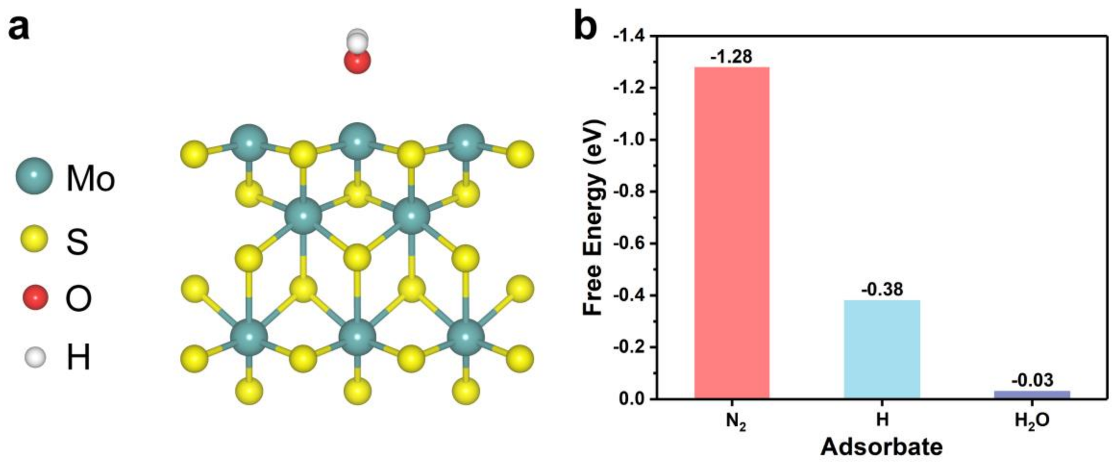

Figure S44. (a) The optimized adsorption geometries of H_2O molecule on the Mo-edge of 1T-MoS_2. (b) The calculated adsorption energy of the different adsorbates at the same site.

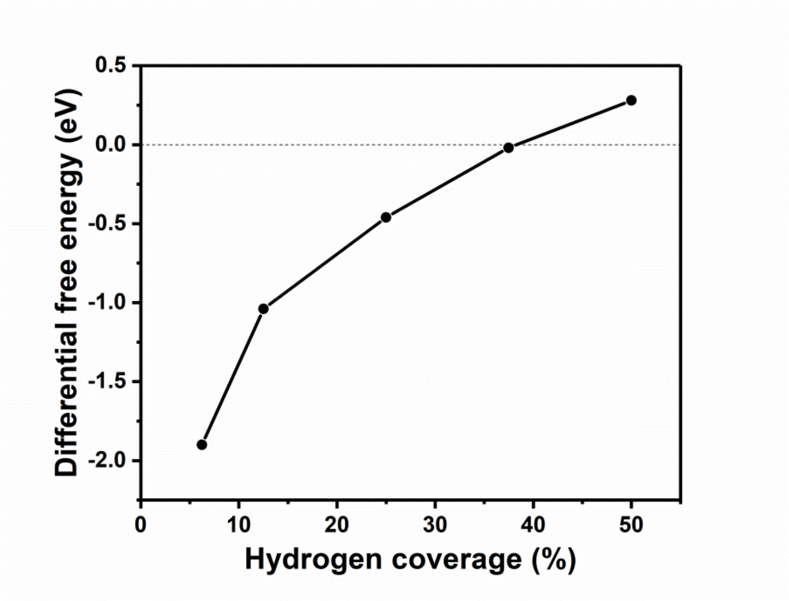

Figure S45. Calculated differential free energy of hydrogen adsorption (ΔG_H) as a function of hydrogen coverage on the basal plane of 1T-MoS_2.

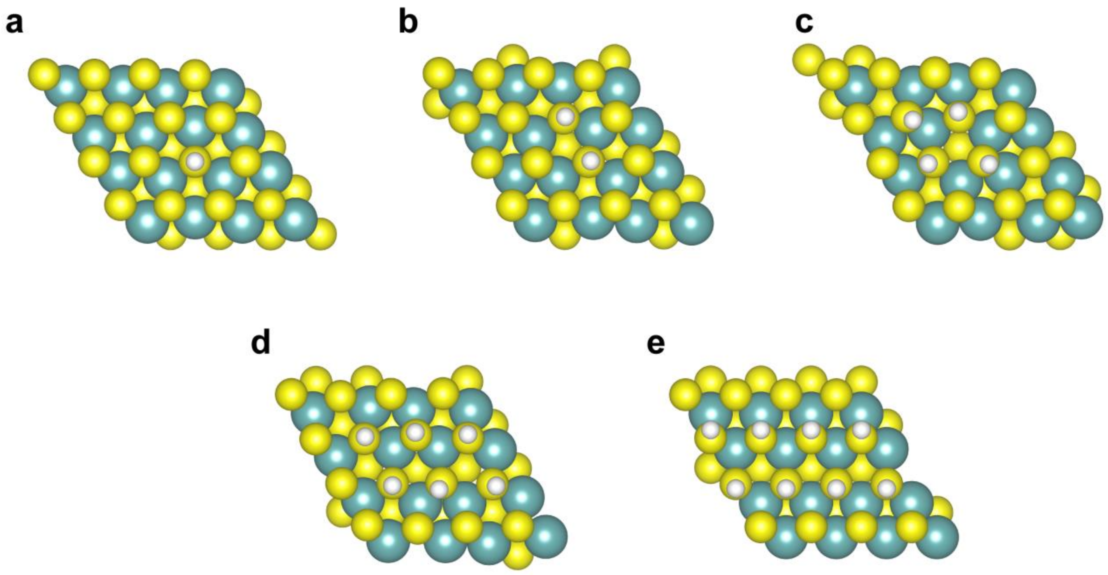

Figure S46. The optimized adsorption geometries of H on 1T-MoS_2 surface at different H coverages: (a) 6.25 %, (b) 12.5 %, (c) 25.0 %, (d) 37.5 %, and (e) 50.0 %.

Reference

[1] X. L. Lu, K. Xu, P. G. Chen, K.C. Jia, S. Liu, C. Z. Wu, *J. Mater. Chem. A* **2014**, *2*, 18924.

[2] G. X. Lin, Q. J. Ju, X. W. Guo, W. Zhao, S. Adimi, J. Y. Ye, Q. Y. Bi, J. C. Wang, M. H. Yang, F. Q. Huang, *Adv. Mater.* **2021**, *2007509*.

[3] Y. C. Hao, Y. Guo, L. W. Chen, M. Shu, X. Y. Wang, T. A. Bu, W. Y. Gao, N. Zhang, X. Su, X. Feng, J. W. Zhou, B. Wang, C. W. Hu, A. X. Yin, R. Si, Y. W. Zhang, C. H. Yan, *Nat. Catal.* **2019**, *2*, 448.

[4] S. Z. Andersen, V. Colic, S. Yang, J. A. Schwalbe, A. C. Nielander, J. M. McEnaney, K. Enemark-Rasmussen, J. G. Baker, A. R. Singh, B. A. Rohr, M. J. Statt, S. J. Blair, S. Mezzavilla, J. Kibsgaard, P. C. K. Vesborg, M. Cargnello, S. F. Bent, T. F. Jaramillo, I. E. L. Stephens, J. K. Norskov, I. Chorkendorff, *Nature* **2019**, *570*, 504.

[5] W. Fang, J. Zhao, T. Wu, Y. Huang, L. Yang, C. Liu, Q. Zhang, K. Huang, Q. Yan, *J. Mater. Chem. A* **2020**, *8*, 5913.

[6] S. Jiao, Z. Yao, F. Xue, Y. Lu, M. Liu, H. Deng, X. Ma, Z. Liu, C. Ma, H. Huang, S. Ruan, Y. Zeng, *Appl. Catal., B* **2019**, *258*, 117964.

[7] Z. Zhao, H. Yang, Y. Zhu, S. Luo, J. Ma, *Nanoscale* **2019**, *11*, 12938.

[8] D. Wang, X. Zhang, S. Bao, Z. Zhang, H. Fei, Z. Z. Wu, *J. Mater. Chem. A* **2017**, *5*, 2681.

[9] C.Y. Ling, Y. X. Ouyang, Q. Li, X.W. Bai, X. Mao, A. J. Du, J. L. Wang, *Small Methods.* **2019**, *3*, 1800376.

[10] K. Mathew, R. Sundararaman, K. L. Weaver, T. A. Arias, R. G. Hennig, *J. Chem. Phys.* **2014**, *140*, 084106.

[11] K. Mathew, V. S. C. Kolluru, S. Mula, S. N. Steinmann, R. G. Hennig, *J. Chem. Phys.* **2019**, *151*, 234101.

[12] N. M. Reynolds, K. J. Kim, C. Chang, S. L. Hsu, *Macromolecules* **1989**, *22*, 1092.

[13] L. Q. Ye, J. Y. Liu, Z. Jiang, T. Y. Peng, L. Zan, *Appl. Catal., B* **2013**, *142-143*, 1.

[14] Y. Zhang, J. S. Liu, X. F. Chu, S. M. Liang, L. B. Kong, *J. Alloys Compd.* **2020**, *832*, 153355.

[15] S. Mahak, A. P. Amol, V. Chandra, *J. Colloid Interface Sci.* **2016**, *464*, 167.

[16] P. Larkin, *Infrared and Raman spectroscopy: principles and spectral interpretation*, Elsevier, San Diego, Oxford **2011**.

[17] Y. Du, H. X. Fang, Q. Zhang, H. L. Zhang, Z. Hong, *Spectrochim. Acta, Part A* **2016**, *153*, 580.

[18] G. D. Jiang, X. X. Yang, Y. Wu, Z. W. Li, Y. H. Han, X. D. Shen, *Mol. Catal.* **2017**, *432*, 232.

[19] A. A. Jeffery, C. Nethravathi, M. Rajamathi, *J. Phys. Chem. C* **2014**, *118*, 1386.

[20] X. S. Xu, X. J. Tian, B. T. Sun, Z. Q. Liang, H. Z. Cui, J. Tian, M. H. Shao, *Appl. Catal., B* **2020**, *272*, 118984.

[21] X. S. Xu, B. T. Sun, Z. Q. Liang, H. Z. Cui, J. Tian, *ACS Appl. Mater. Interfaces* **2020**, *12*, 26060.

[22] H. Mao, Y. L. Fu, H. R. Yang, Z. Z. Deng, Y. Sun, D. L. Liu, Q. Wu, T. Y. Ma, X. M. Song, *ACS Appl. Mater. Interfaces* **2020**, *12*, 25189.

[23] B. H. R. Suryanto, D. B. Wang, L. M. Azofra, M. Harb, L. Cavallo, R. Jalili, R. G. Mitchell, M. Chatti, D. R. MacFarlane, *ACS Energy Lett.* **2019**, *4*, 430.

[24] X. Li, T. Li, Y. Ma, Q. Wei, W. Qiu, H. Guo, X. Shi, P. Zhang, A. M. Asiri, L. Chen, B. Tang, X. Sun, *Adv. Energy Mater.* **2018**, *8*, 1801357.

[25] X. Li, X. Ren, X. Liu, J. Zhao, X. Sun, Y. Zhang, X. Kuang, T. Yan, Q. Wei, D. Wu, *J. Mater. Chem. A* **2019**, *7*, 2524.

[26] L. Zhang, X. Ji, X. Ren, Y. Ma, X. Shi, Z. Tian, A. M. Asiri, L. Chen, B. Tang, X. Sun, *Adv. Mater.* **2018**, *30*, 1800191.

[27] K. Chu, Y. P. Liu, Y. B. Li, Y. L. Guo, Y. Tian, *ACS Appl. Mater. Interfaces* **2020**, *12*, 7081.

[28] J. Zhang, X. Y. Tian, M. J. Liu, H. Guo, J. D. Zhou, Q. Y. Fang, Z. Liu, Q. Wu, J. Lou, *J. Am. Chem. Soc.* **2019**, *141*, 19269.

[29] W. R. Liao, K. Xie, L. J. Liu, X. Y. Wang, Y. Luo, S. J. Liang, F. J. Liu, L. L. Jiang, *J. Energy Chem.* **2021**, *62*, 359.

[30] L. Zeng, S. Chen, J. V. D. Zalm, X. Li, A. Chen, *Chem. Commun.* **2019**, *55*, 7386.

[31] H. Y. Su, L. L. Chen, Y. Z. Chen, Y. T. Wu, X. N. Wu, R. Si, W. H. Zhang, Z. G. Geng, J. Zeng, *Angew. Chem., Int. Ed.* **2020**, *59*, 20411.

[32] D. S. Yang, T. Chen, Z. J. Wang, *J. Mater. Chem. A* **2017**, *5*, 18967.

[33] Y. Y. Ma, T. Yang, H. Y. Zou, W. J. Zang, Z. K. Kou, L. Mao, Y. P. Feng, L. Shen, S. J.

Pennycook, L. Duan, X. Li, J. Wang, *Adv. Mater.* **2020**, *32*, 2002177.

[34] X. Ren, J. Zhao, Q. Wei, Y. Ma, H. Guo, Q. Liu, Y. Wang, G. Cui, A. M. Asiri, B. Li, B. Tang, X. Sun, *ACS Cent. Sci.* **2018**, *5*, 116-121.

[35] H. Cheng, L. X. Ding, G. F. Chen, L. L. Zhang, J. Xue, H. H. Wang, *Adv. Mater.* **2018**, *30*, 1803694.

[36] L. Zhang, X. Ji, X. Ren, Y. Luo, X. Shi, A. M. Asiri, B. Zheng, X. Sun, *ACS Sustainable Chem. Eng.* **2018**, *6*, 9550.

[37] X. Ren, G. Cui, L. Chen, F. Xie, Q. Wei, Z. Tian, X. Sun, *Chem. Commun.* **2018**, *54*, 8474.

[38] J. Han, X. Ji, X. Ren, G. Cui, L. Li, F. Xie, H. Wang, B. Li, X. Sun, *J. Mater. Chem. A* **2018**, *6*, 12974.

[39] W. Kong, R. Zhang, X. Zhang, L. Ji, G. Yu, T. Wang, Y. Luo, X. Shi, Y. Xu, X. Sun, *Nanoscale* **2019**, *11*, 19274.

[40] R. Zhang, H. R. Guo, L. Yang, Y. Wang, Z. G. Niu, H. Huang, H. Y. Chen, L. Xia, T. S. Li, X. F. Shi, X. P. Sun, B. H. Li, Q. Liu, *ChemElectroChem* **2019**, *6*, 1014.

[41] C. Lv, Y. M. Qian, C. S. Yan, Y. Ding, Y. Y. Liu, G. Chen, G. H. Yu, *Angew. Chem., Int. Ed.* **2018**, *57*, 10246.

[42] R. Zhang, J. R. Han, B. Z. Zheng, X. F. Shi, A. M. Asiri, X. P. Sun, *Inorg. Chem. Front.* **2019**, *6*, 391.

[43] X. X. Zhang, Q. Liu, X. F. Shi, A. M. Asiri, Y. L. Luo, X. P. Sun, T. S. Li, *J. Mater. Chem. A* **2018**, *6*, 17303.

[44] Y. Wang, X. Q. Cui, J. X. Zhao, G. R. Jia, L. Gu, Q. H. Zhang, L. K. Meng, Z. Shi, L. R. Zheng, C. Y. Wang, Z. W. Zhang, W. T. Zheng, *ACS Catal.* **2019**, *9*, 336.

[45] J. X. Zhao, L. Zhang, X. Y. Xie, X. H. Li, Y. J. Ma, Q. Liu, *J. Mater. Chem. A* **2018**, *6*, 24031.

[46] W. Z. Fu, Y. D. Cao, Q. Y. Feng, W. R. Smith, P. Dong, M. X. Ye, J. F. Shen, *Nanoscale* **2019**, *11*, 1379.

[47] Y. Zhang, W. Qiu, Y. G. Ma, Y. L. Luo, Z. Q. Tian, G, W. Cui, F. Y. Xie, L. Chen, X. P. Sun, *ACS Catal.* **2018**, *8*, 8540.

[48] C. He, Z. Y. Wu, L. Zhao, M. Ming, Y. Zhang, Y. Yi, J. S. Hu, *ACS Catal.* **2019**, *9*, 7311.

[49] H. Huang, L. Xia, R. R. Cao, Z. G. Niu, H. Y. Chen, Q. Liu, T. S. Li, X. F. Shi, A. M. Asiri, X. P. Sun, *Chem. - Eur. J.* **2018**, *25*, 1914.

[50] J. Wang, L. Yu, L. Hu, G. Chen, H. L. Xin, X. F. Feng, *Nat. Commun.* **2018**, *9*, 1795.
